# Synthesis and in vitro antimicrobial activity screening of new pipemidic acid derivatives

**DOI:** 10.1007/s12272-018-1025-3

**Published:** 2018-04-04

**Authors:** Łukasz Popiołek, Anna Biernasiuk, Kinga Paruch, Anna Malm, Monika Wujec

**Affiliations:** 1 Department of Organic Chemistry, Faculty of Pharmacy, Medical University of Lublin, 4A Chodźki Street, 20-093 Lublin, Poland; 2 Department of Pharmaceutical Microbiology, Faculty of Pharmacy, Medical University of Lublin, 1 Chodźki Street, 20-093 Lublin, Poland

**Keywords:** Pipemidic acid, Mannich bases, Antimicrobial activity, MIC, MBC

## Abstract

**Abstract:**

This article describes the synthesis and antimicrobial activity evaluation of new pipemidic acid derivatives. New compounds were obtained on the basis of Mannich reaction of 4,5-disubstituted 1,2,4-triazole-3-thiones with pipemidic acid. Antimicrobial tests revealed high antibacterial activity of obtained derivatives. Gram-negative rods belonging to *Enterobacteriaceae* family were particularly most sensitive to new pipemidic acid derivatives. Synthesized compounds exhibited very strong activity towards *Proteus mirabilis* ATCC 12453, *Salmonella typhimurium* ATCC 14028 and *Escherichia coli* ATCC 25922. The minimum inhibitory concentrations of new pipemidic acid derivatives which inhibited the growth of these bacteria were 0.98–7.81 µg/ml, 0.98–7.81 µg/ml and 0.98–3.91 µg/ml, respectively. The antibacterial activity of newly synthesized pipemidic acid derivatives in many cases was far better than the activity of substances used as positive controls (nitrofurantoin, cefuroxime, ampicillin and pipemidic acid).

**Graphical Abstract:**

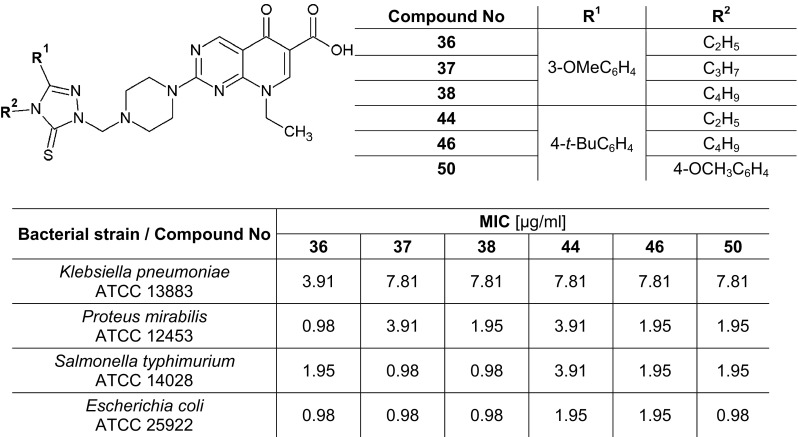

**Electronic supplementary material:**

The online version of this article (10.1007/s12272-018-1025-3) contains supplementary material, which is available to authorized users.

## Introduction

Microorganisms play a key role in the functioning of the environment but they are also a real threat to human life and health. The widespread diseases and infections caused by different bacteria and fungi have encourage the need for scientific research and advances in medicinal chemistry (Coates et al. 2002; Spellberg et al. 2008; Llor and Bjerrum 2014). The unequivocal reports regarding the scale of pathogenic microbial resistance to commonly used drugs have forced the search for new antimicrobial agents (Coates et al. 2002; Spellberg et al. 2008; Llor and Bjerrum 2014).

Literature review prove that researchers around the world are looking for substances that will be better tolerated by patients, less toxic, and at the same time more effective in combating with microbes (Spellberg et al. 2008; Llor and Bjerrum 2014). Many medicinal chemists are concentrating their studies on compounds having heterocyclic systems with nitrogen atoms in their structure (Spellberg et al. 2008; Llor and Bjerrum 2014). Especially, 1,2,4-triazole-3-thiones focused attention of many researchers mainly due to their usefulness in different reactions e.g., Mannich reaction (Bala et al. 2014). Due to the simplicity of the Mannich reaction and favorable pharmacological effect of its products, this reaction is often used in the pharmaceutical industry (Bala et al. 2014).

Mannich bases display wide spectrum of biological activity, like: antibacterial (Pandeya et al. 2000; Ashok et al. 2007), antifungal (Pandeya et al. 2000; Singh et al. 2007), antitubercular (Mulla et al. 2011), antimalarial (Barlin and Jiravinya 1990), anti-HIV (Sriram et al. 2009), anti-inflammatory (Köksal et al. 2007; Ivanova et al. 2007), anti-cancer (Gul et al. 2000), anticonvulsant (Vashishtha et al. 2004), analgesic (Malinka et al. 2005; Köksal et al. 2007), antipsychotic activity (Scott et al. 1992) and activity against herpes virus (Edwards et al. 1983). Additionally, it is worth to mention that in our previous reports Mannich bases derived form 1,2,4-triazole-3-thiones displayed interesting antiproliferative and antimicrobial activity (Popiołek et al. 2014, 2017).

In our current research we focused our attention on both Mannich bases and pipemidic acid. Pipemidic acid is an antimicrobial agent from the quinolones group. Its mechanism of action involves inhibition of DNA synthesis in bacterial cells by blocking class II topoisomerases, namely DNA gyrase which is responsible for DNA stranding and spatial DNA isomer formation, and topoisomerase I responsible for DNA strand separation after replication process. Inhibition of the activity of these enzymes leads to the damage of bacterial DNA. Inhibition of DNA gyrase results in loosening of the structure and increasing of the space occupied by DNA in the bacterial cell (Shumitzu et al. 1975; Domagala 1994; Hawkey 2003; Andersson and MacGowan 2003). Pipemidic acid is therefore considered to be a bactericidal compound (Shumitzu et al. 1975). From the chemical point of view, pipemidic acid is a pyrimidine derivative active against Gram-negative bacteria, including *Pseudomonas aeruginosa*, as well as some Gram-positive bacteria. Its activity is generally greater than pyrimidine acid and nalidixic acid (Shumitzu et al. 1975). In addition to this, literature findings proved that the connection of 1,3,4-thiadiazoles or 1,2,4-triazoles with (fluoro)quinolones in one molecule may have beneficial influence on the antimicrobial activity of such hybrid compounds (Foroumadi et al. 2006; Plech et al. 2013).

In the view of above mentioned facts in this current research we decided to synthesize novel pipemidic acid derivatives with the use of Mannich reaction of 4,5-disubstituted 1,2,4-triazole-3-thione derivatives with pipemidic acid in believe to obtain compounds with interesting antimicrobial activity.

## Materials and methods

### Chemistry

The reagents and solvents used in this research were obtained from Merck Co. (Darmstadt, Germany) and Sigma-Aldrich (Munich, Germany). Melting points were determined with the use of Fisher-Johns blocks melting point apparatus (Fisher Scientific, Germany) and presented without correction. The ^1^H NMR and ^13^C NMR spectra were recorded with the use of Bruker Avance 300 apparatus (Bruker BioSpin GmbH, Germany). The DMSO-*d*_*6*_ was used as solvent and TMS as the internal standard. Chemical shifts in this article are reported in ppm (δ). The coupling constants (*J*) are presented in Hertz. The purity of obtained compounds and the progress of the reaction were determined by thin-layer chromatography (TLC) with the use of pre-coated aluminum sheet 60 F254 plates (Merck Co. USA), and CHCl_3_/C_2_H_5_OH (10:1, v/v) solvent system. The spots were identified by the exposure to the UV light at 254 nm. The elemental analysis of obtained compounds was carried out with the use of AMZ 851 CHX analyser (PG, Gdańsk, Poland). The results of elemental analysis (C, H, N) were within ± 0.4% of the calculated values.

#### Synthesis of thiosemicarbazide derivatives (**3**–**18**)

0.002 Mole of appropriate carboxylic acid hydrazide (3-methoxybenzhydrazide—**1** or 4-*tert*-butylbenzhydrazide—**2**, respectively) was dissolved in 10 ml of ethanol (96%). Then 0.0022 mol of appropriate isothiocyanate was added and heated under reflux for 3 h. Subsequently obtained solution was put to the refrigerator for 24 h. After that formed precipitate was filtered off and re-crystallized from ethanol. The procedure of this synthesis was based on our previous article (Popiołek et al. 2013a).

Detailed physicochemical data of thiosemicarbazide derivatives (**3**–**18**) is presented in Supplementary Materials.

#### Synthesis of 4,5-disubstituted 1,2,4-triazole-3-thione derivatives (**19**–**34**)

4,5-Disubstituted 1,2,4-triazole-3-thiones were synthesized with the use of the procedure from our earlier research (Popiołek et al. 2013b). 0.003 Mole of appropriate thiosemicarbazide derivative (**3**–**18**) was dissolved in 5 ml of 2% sodium hydroxide solution and heated under reflux for 2 h. Subsequently, obtained solution was neutralized with diluted hydrochloric acid. Formed precipitate was filtered off and re-crystallized from ethanol.

Detailed physicochemical data of 4,5-disubstituted 1,2,4-triazole-3-thione derivatives (**19**–**34**) is presented in Supplementary Materials.

#### Synthesis of new pipemidic acid derivatives (**35**–**50**)

In order to obtained new pipemidic acid derivatives we applied the Mannich reaction and the procedure reported by our group earlier (Popiołek et al. 2014, 2017). 0.003 Mole of appropriated 4,5-disubstituted 1,2,4-triazole-3-thione derivative (**19**–**34**) was added to the conical flask and dissolved with stirring in 5 ml of ethanol (96%). After that 200 µl of formaldehyde and 0.0033 mol of pipemidic acid was added to the flask. The content of the flask was stirred by magnetic stirrer for 1 h. Subsequently, 15 ml of distilled water was added to the flask. The precipitate which formed was filtered off and re-crystallized from methanol.

Detailed physicochemical data of new pipemidic acid derivatives (**35**–**50**) is presented in Supplementary Materials.

### Microbiology

The examined compounds **3**–**50** were screened in vitro for antibacterial and antifungal activities using the broth microdilution method according to European Committee on Antimicrobial Susceptibility Testing (EUCAST) (EUCAST discussion document E. Dis 5.1 2003) and Clinical and Laboratory Standards Institute guidelines (Reference method for broth dilution antifungal susceptibility testing of yeasts. M27-S4 2012) against a panel of reference and clinical or saprophytic strains of microorganisms. Ciprofloxacin, nitrofurantoin, cefuroxime, ampicillin and pipemidic acid (Sigma–Aldrich, Munich, Germany) were used as a reference antibacterial compounds. Nystatin (Sigma–Aldrich, Munich, Germany) was used as reference antifungal positive control. Detailed procedure of antimicrobial screening is presented in Supplementary Materials. The statistical analysis of obtained results is presented in the Tables 1S, 2S, and 3S in Supplementary Materials.

## Results

### Chemistry

**Scheme 1:**
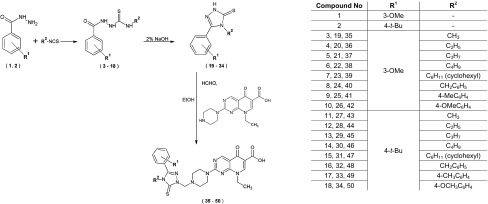
Synthetic route to new pipemidic acid derivatives

New pipemidic acid derivatives (**35**–**50**) were obtained with the use of three step reaction scheme (Scheme 1). Firstly, thiosemicarbazide derivatives (**3**–**18**) were synthesized on the basis of condensation reaction of appropriate carboxylic acid hydrazides with various isothiocyanates. Subsequently, thiosemicarbazide derivatives (**3**–**18**) underwent cyclization reaction with the use of 2% sodium hydroxide solution, which afforded the 4,5-disubstituted 1,2,4-triazole-3-thione derivatives (**19**–**34**). Finally, 1,2,4-triazole-3-thione derivatives (**19**–**34**) were subjected to Mannich reaction with pipemidic acid to obtain new pipemidic acid derivatives (**35**–**50**). Chemical structure of all obtained compounds (**3**–**50**) was confirmed on the basis of spectral identification and elemental analysis.

### Microbiology

All synthesized compounds, including thiosemicarbazides (**3**–**18**), 1,2,4-triazoles-3-thiones (**19**–**34**) and pipemidic acid derivatives (**35**–**50**), were in vitro screened against references strains of bacteria and fungi. The results of antimicrobial assays are presented in the Tables 1, 2 and 3.

**Table 1:** The activity data of compounds **3**–**18** expressed as MIC (MBC or MFC) [µg/ml] and {MBC/MIC or MFC/MIC} against the reference strains of bacteria and fungi

Species	MIC (MBC/MFC) [µg/ml] and {MBC/MIC or MFC/MIC} of the tested compounds					MIC (MBC) [µg/ml] of reference substances				
						CIP/NY*	NIT	CFX	APC	PA
	**3**	**11**	**14**	**16**	**17**					
Gram-positive bacteria										
*Staphylococcus aureus* ATCC 25923	–	1000 (> 1000) {nd}	–	1000 (> 1000) {nd}	250 (> 1000) {nd}	0.488	15.62	0.49	nd	31.25 (62.5)
*Staphylococcus aureus* ATCC 6538	–	1000 (> 1000) {nd}	–	1000 (> 1000) {nd}	125 (> 1000) {nd}	0.244	15.62	0.98	nd	62.5 (125)
*Staphylococcus aureus* ATCC 43300	1000 (> 1000) {nd}	–	–	–	1000 (> 1000) {nd}	0.244	7.81	nd	nd	62.5 (125)
*Staphylococcus epidermidis* ATCC 12228	–	–	–	–	250 (> 1000) {nd}	0.122	3.91	0.24	nd	15.62 (62.5)
*Micrococcus luteus* ATCC 10240	–	1000 (> 1000) {nd}	–	1000 (> 1000) {nd}	3.91 (500) {128}	0.976	62.5	0.98	nd	125 (250)
*Bacillus subtilis* ATCC 6633	–	–	1000 (> 1000) {nd}	–	31.25 (> 1000) {nd}	0.031	3.91	15.62	nd	3.91 (7.81)
*Bacillus cereus* ATCC 10876	–	–	–	–	7.81 (1000) {128}	0.061	7.81	31.25	62.5	15.62 (62.5)
Gram-negative bacteria										
*Bordetella bronchiseptica* ATCC 4617	1000 (> 1000) {nd}	500 (> 1000) {nd}	–	–	500 (> 1000) {nd}	0.976	125	nd	nd	31.25 (62.5)
*Klebsiella Pneumoniae* ATCC 13883	–	–	–	–	1000 (> 1000) {nd}	0.122	15.62	nd	nd	15.62 (31.25)
*Proteus mirabilis* ATCC 12453	–	–	–	–	–	0.030	62.5	nd	nd	1.95 (3.91)
*Salmonella typhimurium* ATCC 14028	–	–	–	–	1000 (> 1000) {nd}	0.061	31.25	nd	nd	1.95 (3.91)
*Escherichia coli* ATCC 25922	–	–	–	–	1000 (> 1000) {nd}	0.004	7.81	nd	nd	0.98 (1.95)
*Pseudomonas aeruginosa* ATCC 9027	–	–	–	–	–	0.488	nd	nd	nd	31.25 (62.5)
Fungi										
*Candida albicans* ATCC 2091	**1000** **(1000)** **{1}**	**250** **(500)** **{2}**	–	–	500 (> 1000) {nd}	0.24*	na	na	na	na
*Candida albicans* ATCC 10231	**1000** **(1000)** **{1}**	**250** **(500)** **{2}**	–	–	500 (1000) {2}	0.48*	na	na	na	na
*Candida parapsilosis* ATCC 22019	**1000** **(1000)** **{1}**	**250** **(500)** **{2}**	–	–	250 (1000) {4}	0.24*	na	na	na	na
*Candida glabrata* ATCC 90030	1000 (1000) {nd}	**1000** **(1000)** **{1}**	–	–	1000 (1000) {nd}	0.24*	na	na	na	na
*Candida krusei* ATCC 14243	1000 (1000) {nd}	**250** **(500)** **{2}**	–	–	1000 (1000) {nd}	0.24*	na	na	na	na

The standard compounds used as positive controls: ciprofloxacin (CIP), nitrofurantoin (NIT), cefuroxime (CFX), ampicillin (APC) and pipemidic acid (PA) for bacteria and nystatin (NY*) for fungi. Compounds with bactericidal effect (MBC/MIC ≤ 4) are marked in bold. All the experiments were repeated three times (n = 3) and representative data is presented

*nd* not determined, *na* not applicable, “–” no activity

**Table 2:** The activity data of compounds **19**–**34** expressed as MIC (MBC or MFC) [µg/ml] and {MBC/MIC or MFC/MIC} against the reference strains of bacteria and fungi

Species	MIC (MBC/MFC) [µg/ml] and {MBC/MIC or MFC/MIC} of the tested compounds				MIC (MBC) [µg/ml] of reference substances				
					CIP/NY*	NIT	CFX	APC	PA
	**19**	**20**	**21**	**34**					
Gram-positive bacteria									
*Staphylococcus aureus* ATCC 25923	1000 (> 1000) {nd}	1000 (> 1000) {nd}	500 (> 1000) {nd}	1000 (> 1000) {nd}	0.488	15.62	0.49	nd	31.25 (62.5)
*Staphylococcus aureus* ATCC 6538	1000 (> 1000) {nd}	–	500 (> 1000) {nd}	500 (> 1000) {nd}	0.244	15.62	0.98	nd	62.5 (125)
*Staphylococcus aureus* ATCC 43300	1000 (> 1000) {nd}	–	1000 (> 1000) {nd}	1000 (> 1000) {nd}	0.244	7.81	nd	nd	62.5 (125)
*Staphylococcus epidermidis* ATCC 12228	1000 (> 1000) {nd}	–	1000 (> 1000) {nd}	1000 (> 1000) {nd}	0.122	3.91	0.24	nd	15.62 (62.5)
*Micrococcus luteus* ATCC 10240	500 (> 1000) {nd}	1000 (> 1000) {nd})	500 (> 1000) {nd}	500 (> 1000) {nd}	0.976	62.5	0.98	nd	125 (250)
*Bacillus subtilis* ATCC 6633	500 (> 1000) {nd}	–	**500** **(1000)** **{2}**	1000 (> 1000) {nd}	0.031	3.91	15.62	nd	3.91 (7.81)
*Bacillus cereus* ATCC 10876	1000 (> 1000) {nd}	1000 (> 1000) {nd}	500 (> 1000) {nd}	1000 (> 1000) {nd}	0.061	7.81	31.25	62.5	15.62 (62.5)
Gram-negative bacteria									
*Bordetella bronchiseptica* ATCC 4617	–	1000 (> 1000) {nd}	1000 (> 1000) {nd}	–	0.976	125	nd	nd	31.25 (62.5)
*Klebsiella pneumoniae* ATCC 13883	–	–	–	–	0.122	15.62	nd	nd	15.62 (31.25)
*Proteus mirabilis* ATCC 12453	–	–	–	–	0.030	62.5	nd	nd	1.95 (3.91)
*Salmonella typhimurium* ATCC 14028	–	–	–	–	0.061	31.25	nd	nd	1.95 (3.91)
*Escherichia coli* ATCC 25922	–	–	–	–	0.004	7.81	nd	nd	0.98 (1.95)
*Pseudomonas aeruginosa* ATCC 9027	–	–	–	–	0.488	nd	nd	nd	31.25 (62.5)
Fungi									
*Candida albicans* ATCC 2091	**250** **(1000)** **{4}**	**250** **(1000)** **{4}**	500 (> 1000) {nd}	–	0.24*	na	na	na	na
*Candida albicans* ATCC 10231	**250** **(1000)** **{4}**	**250** **(1000)** **{4}**	500 (> 1000) {nd}	–	0.48*	na	na	na	na
*Candida parapsilosis* ATCC 22019	**500** **(1000)** **{2}**	**500** **(1000)** **{2}**	1000 (> 1000) {nd}	–	0.24*	na	na	na	na
*Candida glabrata* ATCC 90030	500 (> 1000) {nd}	1000 (> 1000) {nd}	1000 (> 1000) {nd}	–	0.24*	na	na	na	na
*Candida krusei* ATCC 14243	1000 (> 1000) {nd}	1000 (> 1000) {nd}	1000 (> 1000) {nd}	–	0.24*	na	na	na	na

The standard compounds used as positive controls: ciprofloxacin (CIP), nitrofurantoin (NIT), cefuroxime (CFX), ampicillin (APC) and pipemidic acid (PA) for bacteria and nystatin (NY*) for fungi. Compounds with bactericidal effect (MBC/MIC ≤ 4) are marked in bold. All the experiments were repeated three times (n = 3) and representative data is presented

*nd* not determined, *na* not applicable, “–” no activity

**Table 3:** The activity data of compounds **35**–**50** expressed as MIC (MBC or MFC) [µg/ml] and {MBC/MIC or MFC/MIC} against the reference strains of bacteria and fungi

Species	MIC (MBC/MFC) [µg/ml] and {MBC/MIC or MFC/MIC} of the tested compounds										
	**35**	**36**	**37**	**38**	**39**	**40**	**41**	**42**	**43**	**44**	**45**
Gram-positive bacteria											
*Staphylococcus aureus* ATCC 25923	**31.25** **(62.5)** **{2}**	**31.25** **(62.5)** **{2}**	**62.5** **(62.5)** **{1}**	**31.25** **(62.5)** **{2}**	**62.5** **(125)** **{2}**	62.5 (1000) {16}	**62.5** **(125)** **{2}**	125 (> 1000) {nd}	**125** **(250)** **{2}**	**31.25** **(62.5)** **{2}**	**31.25** **(62.5)** **{2}**
*Staphylococcus aureus* ATCC 6538	**31.25** **(31.25)** **{1}**	**15.62** **(31.25)** **{2}**	31.25 (250) {8}	31.25 (250) {8}	**62.5** **(125)** **{2}**	**62.5** **(125)** **{2}**	**62.5** **(125)** **{2}**	**125** **(250)** **{2}**	**125** **(250)** **{2}**	**31.25** **(31.25)** **{1}**	**31.25** **(62.5)** **{2}**
*Staphylococcus aureus* ATCC 43300	**500** **(500)** **{1}**	**500** **(1000)** **{2}**	**500** **(1000)** **{2}**	**500** **(1000)** **{2}**	125 (> 1000) {nd}	500 (> 1000) {nd}	500 (> 1000) {nd}	250 (> 1000) {nd}	**500** **(1000)** **{2}**	**500** **(1000)** **{2}**	**500** **(1000)** **{2}**
*Staphylococcus epidermidis* ATCC 12228	**31.25** **(62.5)** **{2}**	**15.62** **(31.25)** **{2}**	**31.25** **(125)** **{4}**	**31.25** **(31.25)** **{1}**	**31.25** **(125)** **{4}**	**31.25** **(125)** **{4}**	**31.25** **(62.5)** **{2}**	**125** **(125)** **{1}**	**62.5** **(125)** **{2}**	**31.25** **(62.5)** **{2}**	**31.25** **(62.5)** **{2}**
*Micrococcus luteus* ATCC 10240	**250** **(1000)** **{4}**	**250** **(1000)** **{4}**	250 (> 1000) {nd}	250 (> 1000) {nd}	250 (> 1000) {nd}	500 (> 1000) {nd}	250 (> 1000) {nd}	250 (> 1000) {nd}	250 (> 1000) {nd}	250 (> 1000) {nd}	250 (> 1000) {nd}
*Bacillus subtilis* ATCC 6633	**7.81** **(7.81)** **{1}**	**3.91** **(3.91)** **{1}**	**3.91** **(15.62)** **{4}**	**7.81** **(7.81)** **{1}**	**7.81** **(31.25)** **{4}**	7.81 (250) {32}	**7.81** **(15.62)** **{2}**	7.81 (250) {32}	**15.62** **(15.62)** **{1}**	7.81 (250) {32}	**3.91** **(15.62)** **{4}**
*Bacillus cereus* ATCC 10876	15.62 (250) {16}	15.62 (125) {8}	7.81 (500) {64}	15.62 (250) {16}	15.62 (250) {16}	31.25 (250) {16}	15.62 (500) {32}	62.5 (500) {8}	62.5 (500) {8}	31.25 (250) {8}	15.62 (250) {16}
Gram-negative bacteria											
*Bordetella bronchiseptica* ATCC 4617	**31.25** **(62.5)** **{2}**	**31.25** **(31.25)** **{1}**	**31.25** **(31.25)** **{1}**	**31.25** **(62.5)** **{2}**	**62.5** **(125)** **{2}**	**62.5** **(125)** **{2}**	**62.5** **(62.5)** **{1}**	**125** **(125)** **{1}**	**125** **(125)** **{1}**	**31.25** **(62.5)** **{2}**	**31.25** **(31.25)** **{1}**
*Klebsiella pneumoniae* ATCC 13883	**15.62** **(15.62)** **{1}**	**3.91** **(3.91)** **{1}**	**7.81** **(7.81)** **{1}**	**7.81** **(7.81)** **{1}**	**15.62** **(31.25)** **{2}**	**31.25** **(31.25)** **{1}**	**31.25** **(31.25)** **{1}**	**62.5** **(62.5)** **{1}**	**62.5** **(62.5)** **{1}**	**7.81** **(7.81)** **{1}**	**15.62** **(15.62)** **{1}**
*Proteus mirabilis* ATCC 12453	**1.95** **(1.95)** **{1}**	**0.98** **(1.95)** **{2}**	**3.91** **(3.91)** **{1}**	**1.95** **(1.95)** **{1}**	**1.95** **(3.91)** **{2}**	**3.91** **(7.81)** **{2}**	**3.91** **(3.91)** **{1}**	**7.81** **(7.81)** **{1}**	**7.81** **(7.81)** **{1}**	**3.91** **(3.91)** **{1}**	**3.91** **(3.91)** **{1}**
*Salmonella typhimurium* ATCC 14028	**1.95** **(1.95)** **{1}**	**1.95** **(1.95)** **{1}**	**0.98** **(0.98)** **{1}**	**0.98** **(1.95)** **{2}**	**3.91** **(3.91)** **{1}**	**3.91** **(3.91)** **{1}**	**3.91** **(3.91)** **{1}**	**7.81** **(7.81)** **{1}**	**3.91** **(3.91)** **{1}**	**3.91** **(3.91)** **{1}**	**1.95** **(1.95)** **{1}**
*Escherichia coli* ATCC 25922	**1.95** **(1.95)** **{1}**	**0.98** **(0.98)** **{1}**	**0.98** **(0.98)** **{1}**	**0.98** **(0.98)** **{1}**	**1.95** **(3.91)** **{2}**	**3.91** **(3.91)** **{1}**	**1.95** **(3.91)** **{2}**	**3.91** **(3.91)** **{1}**	**3.91** **(3.91)** **{1}**	**1.95** **(3.91)** **{2}**	**0.98** **(0.98)** **{1}**
*Pseudomonas aeruginosa* ATCC 9027	**31.25** **(62.5)** **{2}**	**31.25** **(62.5)** **{2}**	**31.25** **(62.5)** **{2}**	**31.25** **(62.5)** **{2}**	**62.5** **(250)** **{4}**	**125** **(125)** **{1}**	**62.5** **(250)** **{4}**	**125** **(250)** **{2}**	**125** **(250)** **{2}**	**31.25** **(62.5)** **{2}**	**62.5** **(62.5)** **{1}**
Fungi											
*Candida albicans* ATCC 2091	–	–	–	1000 (> 1000) {nd}	–	–	–	1000 (> 1000) {nd}	1000 (> 1000) {nd}	–	–
*Candida albicans* ATCC 10231	–	–	–	1000 (> 1000) {nd}	–	–	–	1000 (> 1000) {nd}	1000 (> 1000) {nd}	–	–
*Candida parapsilosis* ATCC 22019	–	–	–	–	–	–	1000 (> 1000) {nd}	500 (> 1000) {nd}	–	–	–
*Candida glabrata* ATCC 90030	–	–	–	–	–	–	1000 (> 1000) {nd}	1000 (> 1000) {nd}	–	–	–
*Candida krusei* ATCC 14243	–	–	–	–	–	–	–	1000 (> 1000) {nd}	–	–	–

Species	MIC (MBC/MFC) [µg/ml] and {MBC/MIC or MFC/MIC} of the tested compounds					MIC (MBC) [µg/ml] of reference substances				
	**46**	**47**	**48**	**49**	**50**	CIP/NY*	NIT	CFX	APC	PA
Gram-positive bacteria										
*Staphylococcus aureus* ATCC 25923	**31.25** **(62.5)** **{2}**	125 (1000) {8}	62.5 (1000) {16}	**125** **(250)** **{2}**	**31.25** **(62.5)** **{2}**	0.488	15.62	0.49	nd	31.25 (62.5)
*Staphylococcus aureus* ATCC 6538	**31.25** **(62.5)** **{2}**	62.5 (500) {8}	**62.5** **(125)** **{2}**	**62.5** **(250)** **{4}**	**15.62** **(31.25)** **{2}**	0.244	15.62	0.98	nd	62.5 (125)
*Staphylococcus aureus* ATCC 43300	500 (> 1000) {nd}	1000 (> 1000) {nd}	500 (> 1000) {nd}	500 (> 1000) {nd}	250 (> 1000) {nd}	0.244	7.81	nd	nd	62.5 (125)
*Staphylococcus epidermidis* ATCC 12228	**15.62** **(31.25)** **{2}**	**62.5** **(125)** **{2}**	**31.25** **(62.5)** **{2}**	**62.5** **(250)** **{4}**	**7.81** **(31.25)** **{4}**	0.122	3.91	0.24	nd	15.62 (62.5)
*Micrococcus luteus* ATCC 10240	250 (> 1000) {nd}	**500** **(1000)** **{2}**	250 (> 1000) {nd}	500 (> 1000) {nd}	250 (> 1000) {nd}	0.976	62.5	0.98	nd	125 (250)
*Bacillus subtilis* ATCC 6633	**7.81** **(7.81)** **{1}**	7.81 (62.5) {8}	7.81 (62.5) {8}	**31.25** **(31.25)** **{1}**	**3.91** **(7.81)** **{2}**	0.031	3.91	15.62	nd	3.91 (7.81)
*Bacillus cereus* ATCC 10876	15.62 (250) {16}	62.5 (500) {8}	31.25 (500) {8}	62.5 (500) {8}	15.62 (250) {16}	0.061	7.81	31.25	62.5	15.62 (62.5)
Gram-negative bacteria										
*Bordetella bronchiseptica* ATCC 4617	**31.25** **(31.25)** **{1}**	**125** **(125)** **{1}**	**62.5** **(125)** **{2}**	**125** **(250)** **{2}**	**31.25** **(31.25)** **{1}**	0.976	125	nd	nd	31.25 (62.5)
*Klebsiella pneumoniae* ATCC 13883	**7.81** **(7.81)** **{1}**	**62.5** **(62.5)** **{1}**	**31.25** **(62.5)** **{2}**	**62.5** **(62.5)** **{1}**	**7.81** **(7.81)** **{1}**	0.122	15.62	nd	nd	15.62 (31.25)
*Proteus mirabilis* ATCC 12453	**1.95** **(1.95)** **{1}**	**7.81** **(7.81)** **{1}**	**3.91** **(3.91)** **{1}**	**7.81** **(15.62)** **{2}**	**1.95** **(3.91)** **{2}**	0.030	62.5	nd	nd	1.95 (3.91)
*Salmonella typhimurium* ATCC 14028	**1.95** **(1.95)** **{1}**	**3.91** **(7.81)** **{2}**	**3.91** **(7.81)** **{2}**	**7.81** **(7.81)** **{1}**	**1.95** **(3.91)** **{2}**	0.061	31.25	nd	nd	1.95 (3.91)
*Escherichia coli* ATCC 25922	**1.95** **(1.95)** **{1}**	**3.91** **(3.91)** **{1}**	**1.95** **(3.91)** **{2}**	**7.81** **(7.81)** **{1}**	**0.98** **(1.95)** **{2}**	0.004	7.81	nd	nd	0.98 (1.95)
*Pseudomonas aeruginosa* ATCC 9027	**31.25** **(62.5)** **{2}**	**125** **(250)** **{2}**	**62.5** **(125)** **{2}**	**125** **(250)** **{2}**	**31.25** **(62.5)** **{2}**	0.488	nd	nd	nd	31.25 (62.5)
Fungi										
*Candida albicans* ATCC 2091	–	–	–	–	–	0.24*	na	na	na	na
*Candida albicans* ATCC 10231	–	–	–	–	–	0.48*	na	na	na	na
*Candida parapsilosis* ATCC 22019	–	–	–	1000 (> 1000) {nd}	–	0.24*	na	na	na	na
*Candida glabrata* ATCC 90030	–	–	–	–	–	0.24*	na	na	na	na
*Candida krusei* ATCC 14243	–	–	–	–	–	0.24*	na	na	na	na

The standard compounds used as positive controls: ciprofloxacin (CIP), nitrofurantoin (NIT), cefuroxime (CFX), ampicillin (APC) and pipemidic acid (PA) for bacteria and nystatin (NY*) for fungi. Compounds with bactericidal effect (MBC/MIC ≤ 4) are marked in bold. All the experiments were repeated three times (n = 3) and representative data is presented

*nd* not determined, *na* not applicable, “–” no activity

The antibacterial activity of thiosemicarbazide derivatives (**3**–**18**) defined as MIC (Minimal Inhibitory Concentration) values against Gram-positive bacterial strains ranged from 3.91 µg/ml to 1000 µg/ml (MBC—Minimal Bactericidal Concentration = 500 to > 1000 µg/ml), against Gram-negative bacterial strains ranged from 500 µg/ml to 1000 µg/ml (MBC = > 1000 µg/ml). The MIC values against reference fungi were MIC = 250–1000 µg/ml (MFC—Minimal Fungicidal Concentration: 500 to > 1000 µg/ml) (Table 1).

Whereas MIC values of 4,5-disubstituted 1,2,4-triazoles-3-thiones (**19**–**34**) were within the range of 500–1000 µg/ml against Gram-positive bacterial strains (MBC = 1000 to > 1000 µg/ml), 1000 µg/ml against Gram-negative bacterial strains (MBC = > 1000 µg/ml) and 250–1000 µg/ml for fungal strains (MBC = 1000 to > 1000 µg/ml) (Table 2).

Finally, the minimal inhibitory concentrations values for pipemidic acid derivatives (**35**–**50**) ranged from 3.91 to 1000 µg/ml against Gram-positive bacteria (MBC = 3.91 to > 1000 µg/ml), 0.98–125 µg/ml against Gram-negative bacteria (MBC = 0.98–250 µg/ml) and 1000 µg/ml towards fungi belonging to *Candida* spp. (MBC = > 1000 µg/ml) (Table 3).

## Discussion

### Chemistry

The pathway for the synthesis of new pipemidic acid derivatives (Scheme 1) was designed on the basis of literature findings concerning the connection of thiadiazoles or triazoles with (fluoro)quinolones (Foroumadi et al. 2006; Plech et al. 2013) and on our previous reports concerning the synthesis and antimicrobial activity of thiosemicarbazides, 4,5-disubstituted 1,2,4-triazoles-3-thiones as well as Mannich bases derivatives (Popiołek et al. 2013a, b, 2014, 2017). The successful synthesis of thiosemicarbazides (**3**–**18**), 4,5-disubstituted 1,2,4-triazoles-3-thiones (**19**–**34**) and pipemidic acid derivatives (**35**–**50**) in this research was confirmed on the basis of spectral (^1^H NMR and ^13^C NMR) and elemental analysis.

Thiosemicarbazide derivatives (**3**–**18**) showed three typical singlet signals at δ 7.99–10.52 ppm, on the ^1^H NMR spectra, which correspond to three NH groups. Whereas on the ^13^C NMR spectra of this group of compounds (**3**–**18**), signals for carbonyl group (C=O) and thiocarbonyl group (C=S) were found at δ 155.1–159.5 ppm and around δ 166.0 ppm, respectively.

In the case of 4,5-disubstituted 1,2,4-triazole-3-thione derivatives (**19**–**34**), on the ^1^H NMR spectra, singlet signal for NH group was noticed in the range of δ 13.75–14.15 ppm. On the ^13^C NMR spectra the signal for thiocarbonyl group (C=S) of 1,2,4-triazole-3-thiones (**19**–**34**) was found at δ 166.5–169.3 ppm.

The ^1^H NMR spectra of new pipemidic acid derivatives (**35**–**50**) showed characteristic singlet signal for CH_2_ group in the range of δ 5.18–5.36 ppm, what confirmed common aminomethylation reaction of 1,2,4-triazole-3-thiones. Signals for other aliphatic and aromatic fragments of synthesized compounds on ^1^H NMR and ^13^C NMR were shown at expected shift range.

It is worth to mention that all sixteen synthesized pipemidic acid derivatives (**35**–**50**) and six compounds among 4,5-disubstituted 1,2,4-triazole derivatives (**19**–**34**) are new in the literature and their synthesis, physicochemical data and biological activity have not been reported so far.

### Microbiology

Our antimicrobial activity screening results indicated, that most of synthesized compounds among thiosemicarbazide derivatives (**3**–**18**), especially compounds: **4**–**10**, **12**, **13**, **15**, **18**, had no activity against all reference microorganisms (Table 1). Among remaining substances the compound **17** indicated the highest activity with strong and very strong bacteriostatic effect against *Micrococcus luteus* ATCC 10240 and both of *Bacillus* spp. ATCC strains. The minimum concentrations of **17**, which inhibited the growth of these bacteria were 3.91–31.25 and 500 to > 1000 µg/ml, respectively. The activity of the compound **17**, on the basis of minimal inhibitory concentration (MIC) values, was 32 times better against *M. luteus* ATCC 10240 (MIC = 3.91 µg/ml) and two times better against *B. cereus* ATCC 10876 (MIC = 7.81 µg/ml) in comparison with the activity of pipemidic acid (MIC = 125 µg/ml and MIC = 15.62 µg/ml, respectively), used as positive control (Table 1). The compound **17** also showed good bacteriostatic activity towards *Staphylococcus aureus* ATCC 6538 (MIC = 125 µg/ml and MBC > 1000 µg/ml) and moderate or mild effect against other reference staphylococci and some Gram-negative rods (MIC = 250–1000 µg/ml and MBC > 1000 µg/ml). Moreover, the compounds **3**, **11**, **14** and **16** exhibited mild activity (MIC = 1000 µg/ml and MBC > 1000 µg/ml) towards some Gram-positive bacteria. The substances **3** and **11** showed additional effect against *Bordetella bronchiseptica* ATCC 4617 (MIC = 500–1000 µg/ml and MBC > 1000 µg/ml) (Table 1).

Besides this, the compounds **11** and **17** indicated moderate or mild activity towards fungi belonging to all reference yeasts. The minimum concentrations of these substances, which inhibited the growth of *Candida* spp. were from 250 to 1000 µg/ml. The minimal fungicidal concentrations were similar 500 to > 1000 µg/ml. The activity of the compound **3** against yeasts was assessed as mild (MIC = 1000 µg/ml and MFC ≥ 1000 µg/ml) (Table 1).

Among 4,5-disubstituted 1,2,4-triazole-3-thione derivatives (**19**–**34**), only a few, namely **19**, **20**, **21** and **34** exhibited moderate or mild antibacterial activity against some of reference microorganisms (Table 2). The substances **19**, **21** and **34** showed inhibitory effect towards all tested Gram-positive bacteria with MIC = 500–1000 µg/ml and MBC ≥ 1000 µg/ml, while minimum inhibitory concentration of the compound **20** which inhibited the growth of *Staphylococcus aureus* ATCC 25923, *Micrococcus luteus* ATCC 10240 and *Bacillus cereus* ATCC 10876 was 1000 µg/ml and the MBC was > 1000 µg/ml. Moreover, the compounds **20** and **21** indicated activity towards *Bordetella bronchiseptica* ATCC 4617 belonging to Gram-negative bacteria (MIC = 1000 µg/ml and MBC > 1000 µg/ml) (Table 2).

In addition, the compounds **19**, **20** and **21** showed fungicidal activity. The most sensitive to these substances were *Candida albicans* ATCC 2091 and *Candida albicans* ATCC 10231. The minimum inhibitory concentrations of **19**, **20** and **21**, which inhibited their growth were 250–500 µg/ml, while MFC ≥ 1000 µg/ml. These compounds indicated slightly lower effect against other reference species of *Candida* (MIC = 500–1000 µg/ml and MFC ≥ 1000 µg/ml). The remaining substances **22**–**33** were inactive towards bacteria and fungi from ATCC (Table 2).

Newly synthesized pipemidic acid derivatives (**35**–**50**) were highly active against all reference bacteria (Table 3). Gram-negative rods belonging to *Enterobacteriaceae* family were particularly most sensitive to these compounds. All substances (**35**–**50**) showed bactericidal effect against them. These compounds exhibited very strong activity towards *Proteus mirabilis* ATCC 12453, *Salmonella typhimurium* ATCC 14028 and *Escherichia coli* ATCC 25922. The minimum concentrations of **35**–**50** compounds, which inhibited the growth of these bacteria were 0.98–7.81 µg/ml, 0.98–7.81 µg/ml and 0.98–3.91 µg/ml, respectively. *Klebsiella pneumoniae* ATCC 13883 was slightly less susceptible to these substances. The compounds **36**, **37**, **38**, **44**, **46** and **50** indicated very strong activity (MIC = MBC = 3.91–7.81 µg/ml), compounds **35**, **39** and **45**—strong (MIC = 15.62 µg/ml, MBC = 15.62–31.25 µg/ml), while remaining compounds **40**, **41**, **42**, **43**, **47**, **48**, **49**—good activity (MIC = MBC = 31.25–62.5 µg/ml) towards reference *K. pneumoniae* ATCC 13883. In addition, all pipemidic acid derivatives (**35**–**50**) exhibited a similar good effect against *Bordetella bronchiseptica* ATCC 4617 and *Pseudomonas aeruginosa* ATCC 9027 (MIC = MBC = 31.25–125 µg/ml, MBC/MIC = 1–4) (Table 3). Especially, it is worth to underline good activity against *Pseudomonas aeruginosa* ATCC 9027, because this pathogen is responsible for many hospital-acquired and nosocomial infections (Aloush et al. 2006).

In comparison to pipemidic acid, the minimal bactericidal concentration (MBC) values of pipemidic acid derivatives (**35**–**50**) towards *B. bronchiseptica* ATCC 4617 were two times better in case of compounds **36**, **37**, **45**, **46** and **50** (MBC = 31.25 µg/ml). Against *K. pneumoniae* ATCC 13883, the compound **36** showed four times better activity (MIC = 3.91 µg/ml), compounds **37**, **38**, **44**, **46**, and **50** showed two times better activity (MIC = 7.81 µg/ml) than pipemidic acid (MIC = 15.62 µg/ml). The compounds **35** and **45** showed two times lower MBC values (MBC = 15.62 µg/ml) than pipemidic acid (MBC = 31.25 µg/ml) against this bacterium. In the case of the activity against *P. mirabilis* ATCC 12453 the MIC values for the compound **36** were two times lower (MIC = 0.98 µg/ml) and the MBC values for the compounds **35**, **38**, **46** were two times lower (MBC = 1.95 µg/ml) than such values for pipemidic acid (MIC = 1.95 µg/ml, MBC = 3.91 µg/ml). The activity of synthesized derivatives was also better than pipemidic acid towards *Salmonella typhimurium* ATCC 14028. The compounds **37** and **38** showed two times better activity on the basis of MIC values (MIC = 0.98 µg/ml) than pipemidic acid (MIC = 1.95 µg/ml) against this bacterium. The MBC values for compounds **35**, **36**, **45** and **46** (MBC = 1.95 µg/ml) were two times lower than for pipemidic acid used as positive control (MBC = 3.91 µg/ml). In addition to this, the MBC values for compounds **36**, **37**, **38**, and **45** against *E. coli* ATCC 25922 (MBC = 0.98 µg/ml) were two times lower than for pipemidic acid (MBC = 1.95 µg/ml) (Table 3).

The compounds **35**-**50** indicated also high activity towards Gram-positive bacteria but slightly weaker compared to Gram-negative microorganisms. Most of the substances showed very strong bactericidal or bacteriostatic effect against *Bacillus subtilis* ATCC 6633 with MIC = 3.91–7.81 µg/ml, MBC = 3.91–250 µg/ml and MBC/MIC = 1–32. The other compounds **43** and **49** indicated good (MIC = MBC = 31.25 µg/ml) or strong (MIC = MBC = 15.62 µg/ml) bactericidal activity towards this bacterium. *Bacillus cereus* ATCC 10876 was slightly less sensitive to these substances. Among them the compound **37** showed very strong activity (MIC = 7.81 µg/ml, MBC = 500 µg/ml and MBC/MIC = 64) against this bacterium. In turn, substances **35**, **36**, **38**, **39**, **41**, **45**, **46** and **50** had a strong activity (MIC = 15.62 µg/ml, MBC = 125–500 µg/ml and MBC/MIC = 8–32), while compounds **40**, **42**, **43**, **44**, **47**, **48** and **49**—good activity (MIC = 31.25–62.5 µg/ml, MBC = 250–500 µg/ml and MBC/MIC = 8–16) (Table 3).

Pipemidic acid derivatives (**35**–**50**) showed also good activity with bactericidal or bacteriostatic effect against microorganisms belonging to reference staphylococci: *S. aureus* ATCC 25923, *S. aureus* ATCC 6538 and *S. epidermidis* ATCC 12228 (MIC = 31.25–125 µg/ml, MBC = 31.25–1000 µg/ml and MBC/MIC = 1–16 or higher). Among them, compounds **36** and **50** showed strong bactericidal activity towards *S. aureus* ATCC 6538 (MIC = 15.62 µg/ml, MBC = 31.25 µg/ml and MBC/MIC = 2), while compound **50**—very strong (MIC = 7.81 µg/ml, MBC = 31.25 µg/ml, MBC/MIC = 4) and compounds **36** and **46**—strong activity (MIC = 15.62 µg/ml, MBC = 31.25 µg/ml and MBC/MIC = 2) towards *S. epidermidis* ATCC 12228 (Table 3).

Surprisingly, all substances (**35**–**50**) showed only moderate activity towards *Micrococcus luteus* ATCC 10240 (MIC = 250–500 µg/ml and MBC > 1000 µg/ml) and most of them except **39** and **47** against *Staphylococcus aureus* ATCC 43300 (MIC = 250–500 µg/ml and MBC = 500 to > 1000 µg/ml). The compounds **39** and **47** indicated good (MIC = 125 µg/ml and MBC > 1000 µg/ml) or mild (MIC = 1000 µg/ml and MBC > 1000 µg/ml) activity, respectively against *S. aureus* ATCC 43300 (Table 3).

In comparison to the activity of pipemidic used as positive control (MIC = 62.5 µg/ml), the activity of the compounds **36** and **50** (MIC = 15.62 µg/ml) was four times better and for the compounds **35**, **37**, **38**, **44**, **45**, and **46** (MIC = 31.25 µg/ml) was two times better against *S. aureus* ATCC 6538 on the basis of MIC values. The compound **50** showed two times lower MIC values (MIC = 7.81 µg/ml) with bactericidal effect and the compounds **36** and **46** showed two times lower MBC values (MBC = 31.25 µg/ml) with bactericidal effect against *S. epidermidis* ATCC 12228 than pipemidic acid (MIC = 15.62 µg/ml, MBC = 62.5 µg/ml). The MBC values for the derivative **36** towards *B. subtilis* ATCC 6633 (MBC = 3.91 µg/ml) were two times lower than for pipemidic acid (MBC = 7.81 µg/ml). In the case of the activity against *B. cereus* ATCC 10876 the MIC values for the substance **37** were two times lower (MIC = 7.81 µg/ml) than for pipemidic acid (MIC = 15.62 µg/ml) (Table 3). High activity of synthesized compounds (**35**–**50**) against *B. cereus* ATCC 10876 is especially important due to the fact that this bacterium is responsible for an increasing number of foodborne diseases in industrial countries as well as postoperative and posttraumatic wound infections (Kotiranta et al. 2000; Bottone 2010).

Moreover, some of pipemidic acid derivatives (**35**–**50**) indicated also moderate or mild activity towards reference fungi belonging to yeasts. Among them the compound **42** inhibited the growth of all *Candida* spp. (MIC = 500–1000 µg/ml and MFC > 1000 µg/ml). In turn, substances **38**, **41**, **43** and **49** showed mild activity towards some of them (MIC = 1000 µg/ml and MFC > 1000 µg/ml). The remaining compounds were inactive against reference *Candida* spp. (Table 3).

Summarizing, in this research we synthesized and evaluated for in vitro antimicrobial activity a series of new pipemidic acid derivatives obtained by the Mannich reaction of appropriate 4,5-disubstituted 1,2,4-triazole-3-thiones with pipemidic acid. Antimicrobial activity screening of synthesized compounds revealed interesting antibacterial properties of obtained derivatives. Our antimicrobial assays results indicated that newly synthesized pipemidic acid derivatives showed very high antimicrobial activity, especially against Gram-negative bacteria.

## Electronic supplementary material

Below is the link to the electronic supplementary material.

Supplementary material 1 (DOCX 57 kb)
