# Supplementary material for: Synthesis and in vitro antimicrobial activity screening of new pipemidic acid derivatives
Source: Arch Pharm Res. 2018 Apr 4;41(6):633–45. doi: 10.1007/s12272-018-1025-3 (PMC6028826; doi:10.1007/s12272-018-1025-3)
Supplement: Supplementary file 1 — Supplementary material 1 (DOCX 57 kb) [file 12272_2018_1025_MOESM1_ESM.docx]

# Synthesis and *in vitro* antimicrobial activity screening of new pipemidic acid derivatives

# Łukasz Popiołek^1^*, Anna Biernasiuk^2^, Kinga Paruch^1^, Anna Malm^2^, Monika Wujec^1^

^1^ Department of Organic Chemistry, Faculty of Pharmacy,

Medical University of Lublin, 4A Chodźki Street, 20-093 Lublin, Poland,

^2^ Department of Pharmaceutical Microbiology, Faculty of Pharmacy,

Medical University of Lublin, 1 Chodźki Street, 20-093 Lublin, Poland

*e-mail: lukasz.popiolek@umlub.pl

**Supplementary Materials**

***Chemistry***

Detailed physicochemical data of thiosemicarbazide derivatives (**3**-**18**)

2-[(3-Methoxybenzoyl)-*N*-methylhydrazinecarbothioamide (**3**)

CAS Registry Number: 348593-51-1. Yield: 30%; M.p.: 202-204ºC. ^1^H NMR (DMSO-*d_6_*) δ (ppm) = 2.47 (s, 3H, CH_3_), 3.30 (s, 3H, CH_3_), 7.10-7.15 (m, 1H, ArH), 7.36-7.42 (t, 1H, ArH, *J* = 9 Hz), 7.47-7.50 (m, 2H, ArH), 8.02 (s, 1H, NH), 9.29 (s, 1H, NH), 10.29 (s, 1H, NH); ^13^C NMR (DMSO-*d_6_*) δ (ppm) = 30.9 (CH_3_), 56.0 (OCH_3_), 113.3, 118.8, 122.3, 128.5, 133.9, 158.3 (6C_ar_), 159.2 (C=O), 166.6 (C=S); Analysis for C_10_H_13_N_3_O_2_S (239.29) Calculated: C: 50.19%, H: 5.48%, N: 17.56%; Found: C: 50.26%, H: 5.45%, N: 17.64%.

*N*-Ethyl-2-(3-methoxybenzoyl)hydrazinecarbothioamide (**4**)

CAS Registry Number: 712309-59-6. Yield: 87%; M.p.: 180-186ºC. ^1^H NMR (DMSO-*d_6_*) δ (ppm) = 1.04-1.09 (t, 3H, CH_3_, *J* = 6 Hz, *J* = 9 Hz), 3.43-3.50 (q, 2H, CH_2_, *J* = 6 Hz), 7.13-7.16 (m, 1H, ArH), 7.38-7.44 (t, 1H, ArH, *J* = 9 Hz), 7.48-7.52 (m, 2H, ArH), 8.12 (s, 1H, NH), 9.27 (s, 1H, NH), 10.32 (s, 1H, NH); ^13^C NMR (DMSO-*d_6_*) δ (ppm) = 15.0 (CH­_3_), 38.9 (CH_2_), 55.8 (OCH_3_), 113.5, 115.2, 120.5, 129.9, 134.3 (6C_ar_), 159.5 (C=O), 166.1 (C=S); Analysis for C_11_H_15_N_3_O_2_S (253.32) Calculated: C: 52.15%, H: 5.97%, N: 16.59%; Found: C: 52.22%, H: 6.02%, N: 16.55%.

2-(3-Methoxybenzoyl)-*N*-propylhydrazinecarbothioamide (**5**)

CAS Registry Number: 891072-22-3. Yield: 75%; M.p.: 170-172ºC. ^1^H NMR (DMSO-*d_6_*) δ (ppm) = 0.80-0.85 (t, 3H, CH_3_, *J* = 9 Hz, *J* = 6 Hz), 1.45-1.57 (m, 2H, CH_2_), 3.38-3.43 (t, 2H, CH_2_, *J* = 9 Hz, *J* = 6 Hz), 7.12-7.16 (m, 1H, ArH), 7.38-7.43 (t, 1H, ArH, *J* = 9 Hz, *J* = 6 Hz), 7.48-7.52 (m, 2H, ArH), 8.11 (s, 1H, NH), 9.26 (s, 1H, NH), 10.31 (s, 1H, NH); ^13^C NMR (DMSO-*d_6_*) δ (ppm) = 11.7 (CH_3_), 23.1 (CH_2_), 47.2 (CH_2_), 56.0 (OCH_3_), 113.3, 118.9, 122.1, 128.5, 133.9, 159.1 (6C_ar_), 160.1 (C=O), 166.6 (C=S); Analysis for C_12_H_17_N_3_O_2_S (267.35) Calculated: C: 53.91%, H: 6.41%, N: 15.72%; Found: C: 53.87%, H: 6.46%, N: 15.65%.

*N*-Butyl-2-(3-methoxybenzoyl)hydrazinecarbothioamide (**6**)

CAS Registry Number: 905010-52-8. Yield: 59%; M.p.: 140ºC. ^1^H NMR (DMSO-*d_6_*) δ (ppm) = 0.83-0.89 (t, 3H, CH_3_, *J* = 6 Hz), 1.17-1.32 (m, 2H, CH_2_), 1.41-1.52 (m, 2H, CH_2_), 3.38-3.43 (t, 2H, CH_2_, *J* = 6 Hz, *J* = 9 Hz), 7.10-7.15 (m, 1H, ArH), 7.36-7.42 (t, 1H, ArH, *J* = 9 Hz), 7.45-7.50 (m, 2H, ArH), 8.05 (s, 1H, NH), 9.22 (s, 1H, NH), 10.27 (s, 1H, NH); ^13^C NMR (DMSO-*d_6_*) δ (ppm) = 14.3 (CH_3_), 19.9 (CH_2_), 31.4 (CH_2_), 43.8 (CH_2_), 55.8 (OCH_3_), 113.5, 115.1, 120.5, 129.9, 134.3 (6C_ar_), 159.5 (C=O), 166.1 (C=S); Analysis for C_13_H_19_N_3_O_2_S (281.37) Calculated: C: 55.49%, H: 6.81%, N: 14.93%; Found: C: 55.60%, H: 6.77%, N: 14.99%.

*N*-Cyclohexyl-2-(3-methoxybenzoyl)hydrazinecarbothioamide (**7**)

CAS Registry Number: 299923-32-3. Yield: 95%; M.p.: 140-146ºC. ^1^H NMR (DMSO-*d_6_*) δ (ppm) = 1.04-1.35 (m, 5H, CH_2-cyclohexyl_), 1.55-1.78 (m, 5H, CH_2-cyclohexyl_), 3.81 (s, 3H, CH_3_), 4.11-4.15 (m, 1H, CH_cyclohexyl_), 7.13-7.16 (d, 1H, ArH, *J* = 9 Hz), 7.38-7.43 (t, 1H, ArH, *J* = 6 Hz, *J* = 9 Hz), 7.47-7.49 (d, 1H, ArH, *J*= 6 Hz), 7.72-7.75 (d, 1H, ArH, *J* = 9 Hz), 9.21 (s, 1H, NH), 9.77 (s, 1H, NH), 10.26 (s, 1H, NH); ^13^C NMR (DMSO-*d_6_*) δ (ppm) = 25.4 (2xCH_2- cyclohexyl_), 25.6 (2xCH_2-cyclohexyl_), 32.3 (CH_2-cyclohexyl_), 53.5 (CH_cyclohexyl_), 55.8 (OCH_3_), 113.4, 118.0, 120.5, 129.9, 134.3, 135.1 (6C_ar_), 159.5 (C=O), 165.9 (C=S); Analysis for C_15_H_21_N_3_O_2_S (307.41) Calculated: C: 58.61%, H: 6.89%, N: 13.67%; Found: C: 58.52%, H: 6.94%, N: 13.74%.

*N*-Benzyl-2-(3-methoxybenzoyl)hydrazinecarbothioamide (**8**)

CAS Registry Number: 857029-59-5. Yield: 94%; M.p.: 168-172ºC. ^1^H NMR (DMSO-*d_6_*) δ (ppm) = 3.81 (s, 3H, CH_3_), 4.74 (s, 2H, CH_2_), 7.13-7.17 (m, 1H, ArH), 7.21-7.25 (m, 1H, ArH), 7.31-7.33 (m, 4H, ArH), 7.39-7.44 (t, 1H, ArH, *J* = 6 Hz, *J* = 9 Hz), 7.50-7.54 (m, 2H, ArH), 8.68 (s, 1H, NH), 9.49 (s, 1H, NH), 10.43 (s, 1H, NH); ^13^C NMR (DMSO-*d_6_*) δ (ppm) = 47.2 (CH_2_), 55.8 (OCH_3_), 113.6, 118.1, 120.6, 122.5, 127.1, 127.6, 128.5, 129.9, 134.3, 139.9 (12C_ar_), 159.5 (C=O), 166.2 (C=S); Analysis for C_16_H_17_N_3_O_2_S (315.39) Calculated: C: 60.93%, H: 5.43%, N: 13.32%; Found: C: 60.84%, H: 5.39%, N: 13.39%.

2-(3-Methoxybenzoyl)-*N*-(4-methylphenyl)hydrazinecarbothioamide (**9**)

CAS Registry Number: 904981-77-7. Yield: 88%; M.p.: 162-166ºC. ^1^H NMR (DMSO-*d_6_*) δ (ppm) = 2.28 (s, 3H, CH_3_), 3.82 (s, 3H, CH_3_), 7.12-7.17 (m, 3H, ArH), 7.29-7.31 (m, 2H, ArH), 7.39-7.44 (t, 1H, ArH, *J* = 9 Hz), 7.52-7.55 (m, 2H, ArH), 9.66 (s, 1H, NH), 9.76 (s, 1H, NH), 10.52 (s, 1H, NH); ^13^C NMR (DMSO-*d_6_*) δ (ppm) = 21.1 (CH_3_), 56.0 (OCH_3_), 113.3, 119.7, 120.9, 122.1, 128.5, 130.3, 132.7, 133.9, 137.7, 159.1 (12C_ar_), 159.9 (C=O), 166.7 (C=S); Analysis for C_16_H_17_N_3_O_2_S (315.39) Calculated: C: 60.93%, H: 5.43%, N: 13.32%; Found: C: 60.99%, H: 5.47%, N: 13.38%.

2-(3-Methoxybenzoyl)-*N*-(4-methoxyphenyl)hydrazinecarbothioamide (**10**)

CAS Registry Number: 356574-01-1. Yield: 93%; M.p.: 162ºC. ^1^H NMR (DMSO-*d_6_*) δ (ppm) = 3.74 (s, 3H, CH_3_), 3.82 (s, 3H, CH_3_), 6.88-6.91 (d, 2H, ArH, *J* = 9 Hz), 7.13-7.17 (m, 1H, ArH), 7.26-7.29 (d, 2H, ArH, *J* = 9 Hz), 7.39-7.44 (t, 1H, ArH, *J* = 6 Hz, *J* = 9 Hz), 7.52 (s, 1H, ArH), 7.55 (s, 1H, ArH), 9.62 (s, 1H, NH), 9.70 (s, 1H, NH), 10.50 (s, 1H, NH); ^13^C NMR (DMSO-*d_6_*) δ (ppm) = 55.7 (OCH_3_), 55.8 (OCH_3_), 113.7, 118.1, 120.6, 122.2, 128.5, 129.8, 132.5, 134.4, 157.2 (12C_ar_), 159.5 (C=O), 166.2 (C=S); Analysis for C_16_H_17_N_3_O_3_S (331.40) Calculated: C: 57.99%, H: 5.17%, N: 12.68%; Found: C: 57.88%, H: 5.21%, N: 12.76%.

2-(4-*Tert*-butylbenzoyl)-*N*-methylhydrazinecarbothioamide (**11**)

CAS Registry Number: 261705-14-0. Yield: 85%; M.p.: 176-180ºC. ^1^H NMR (DMSO-*d_6_*) δ (ppm) = 1.29 (s, 9H, 3xCH­_3_), 2.84 (s, 3H, CH_3_), 7.47-7.51 (d, 2H, ArH, *J* = 12 Hz), 7.82-7.86 (d, 2H, ArH, *J* = 12 Hz), 7.99 (s, 1H, NH), 9.27 (s, 1H, NH), 10.24 (s, 1H, NH); ^13^C NMR (DMSO-*d_6_*) δ (ppm) = 30.9 (CH_3_), 31.4 (3xCH_3_), 34.3 (C_t-butyl_), 125.9, 130.8, 132.3, 153.4 (6C_ar_), 159.8 (C=O), 166.9 (C=S); Analysis for C_13_H_19_N_3_OS (265.37) Calculated: C: 58.84%, H: 7.22%, N: 15.83%; Found: C: 58.77%, H: 7.30%, N: 15.73%.

2-[(4-*Tert*-butylbenzoyl)-*N*-ethylhydrazinecarbothioamide (**12**)

CAS Registry Number: 405903-89-1. Yield: 95%; M.p.: 176ºC. ^1^H NMR (DMSO-*d_6_*) δ (ppm) = 1.03-1.07 (t, 3H, CH_3_, *J* = 6 Hz), 1.30 (s, 9H, 3xCH_3_), 3.42-3.48 (q, 2H, CH_2_, *J* = 6 Hz), 7.49-7.52 (d, 2H, ArH, *J* = 9 Hz), 7.85-7.88 (d, 2H, ArH, *J* = 9 Hz), 8.07 (s, 1H, NH), 9.25 (s, 1H, NH), 10.26 (s, 1H, NH); ^13^C NMR (DMSO-*d_6_*) δ (ppm) = 14.9 (CH_3_), 31.4 (3xCH_3_), 35.2 (C_­t-butyl­_), 38.9 (CH_2_), 123.8, 125.5, 128.2, 130.2 (6C_ar_), 155.2 (C=O), 166.2 (C=S); Analysis for C_14_H_21_N_3_OS (279.40) Calculated: C: 60.18%, H: 7.58%, N: 15.04%; Found: C: 60.26%, H: 7.49%, N: 15.11%.

2-[(4-*Tert*-butylbenzoyl)-*N*-propylhydrazinecarbothioamide (**13**)

CAS Registry Number: 901358-71-2. Yield: 71%; M.p.: 182ºC. ^1^H NMR (DMSO-*d_6_*) δ (ppm) = 0.80-0.85 (t, 3H, CH_3_, *J* = 6 Hz, *J* = 9 Hz), 1.44-1.56 (m, 2H, CH_2_), 3.37-3.41 (t, 2H, CH_2_, *J* = 6 Hz), 7.49-7.52 (d, 2H, ArH, *J* = 9 Hz), 7.85-7.87 (d, 2H, ArH, *J* = 6 Hz), 8.07 (s, 1H, NH), 9.25 (s, 1H, NH), 10.26 (s, 1H, NH); ^13^C NMR (DMSO-*d_6_*) δ (ppm) = 11.7 (CH_3_), 23.1 (CH_2_), 31.4 (3xCH_3_), 34.3 (C_t-butyl_), 41.2 (CH_2_), 125.9, 130.8, 132.5, 154.1 (6C_ar_), 159.6 (C=O), 166.9 (C=S); Analysis for C_15_H_23_N_3_OS (293.43) Calculated: C: 61.40%, H: 7.90%, N: 14.32%; Found: C: 61.49%, H: 7.83%, N: 14.39%.

*N*-Butyl-2-(4-*tert*-butylbenzoyl)hydrazinecarbothioamide (**14**)

CAS Registry Number: 443637-17-0. Yield: 78%; M.p.: 158-162ºC. ^1^H NMR (DMSO-*d_6_*) δ (ppm) = 0.85-0.90 (t, 3H, CH_3_, *J* = 6 Hz, *J* = 9 Hz), 1.22-1.27 (m, 2H, CH_2_), 1.45-1.52 (m, 2H, CH_2_), 3.39-3.44 (t, 2H, CH_2_, *J* = 9 Hz, *J* = 6 Hz), 7.49-7.52 (d, 2H, ArH, *J* = 9 Hz), 7.85-7.88 (d, 2H, ArH, *J* = 9 Hz), 8.05 (s, 1H, NH), 9.23 (s, 1H, NH), 10.25 (s, 1H, NH); ^13^C NMR (DMSO-*d_6_*) δ (ppm) = 14.0 (CH_3_), 20.2 (CH_2_), 30.9 (CH_2_), 31.4 (3xCH_3_), 34.3 (C_t-butyl_), 44.6 (CH_2_), 125.4, 130.6, 132.1, 154.3 (6C_ar_), 159.3 (C=O), 166.0 (C=S); Analysis for C_16_H_25_N_3_OS (307.45) Calculated: C: 62.50%, H: 8.20%, N: 13.67%; Found: C: 62.60%, H: 8.12%, N: 13.74%.

2-(4-*Tert*-butylbenzoyl)-*N*-cyclohexylhydrazinecarbothioamide (**15**)

CAS Registry Number: 402597-96-0. Yield: 95%; M.p.: 190-192ºC. ^1^H NMR (DMSO-*d_6_*) δ (ppm) = 1.30 (s, 9H, 3xCH_3_), 1.26-1.31 (m, 5H, CH_2-cyclohexyl_), 1.55-1.78 (m, 5H, CH_2-cyclohexyl_), 4.11-4.15 (m, 1H, CH_cyclohexyl_), 7.49-7.52 (d, 2H, ArH, *J* = 9 Hz), 7.85-7.88 (d, 2H, ArH, *J* = 9 Hz), 9.20 (s, 1H, NH), 9.77 (s, 1H, NH), 10.21 (s, 1H, NH); ^13^C NMR (DMSO-*d_6_*) δ (ppm) = 25.4 (2xCH_2-cyclohexyl_), 25.6 (2xCH_2-cyclohexyl_), 31.4 (3xCH_3_), 32.3 (CH_2-cyclohexyl_), 35.1 (C_t-butyl­_), 53.6 (CH_cyclohexyl_), 123.4, 125.5, 128.1, 130.2 (6C_ar_), 155.1 (C=O), 166.1 (C=S); Analysis for C_18_H_27_N_3_OS (333.49) Calculated: C: 64.83%, H: 8.16%, N: 12.60%; Found: C: 64.89%, H: 8.09%, N: 12.68%.

*N*-Benzyl-2-(4-*tert*-butylbenzoyl)hydrazinecarbothioamide (**16**)

CAS Registry Number: 124953-91-9. Yield: 94%; M.p.: 180-186ºC. ^1^H NMR (DMSO-*d_6_*) δ (ppm) = 1.30 (s, 9H, 3xCH_3_), 4.72 (s, 2H, CH_2_), 7.20-7.24 (m, 1H, ArH), 7.30-7.31 (d, 4H, ArH, *J* = 3 Hz), 7.49-7.52 (d, 2H, ArH, *J* = 9 Hz), 7.87-7.89 (d, 2H, ArH, *J* = 6 Hz), 8.64 (s, 1H, NH), 9.46 (s, 1H, NH), 10.37 (s, 1H, NH); ^13^C NMR (DMSO-*d_6_*) δ (ppm) = 31.4 (3xCH_3_), 35.2 (C_t-butyl_), 47.2 (CH_2_), 116.9, 125.5 127.0, 127.6, 128.2, 128.5, 130.1, 139.9 (12C_ar_), 155.2 (C=O), 166.2 (C=S); Analysis for C_19_H_23_N_3_OS (341.47) Calculated: C: 66.83%, H: 6.79%, N: 12.31%; Found: C: 66.89%, H: 6.70%, N: 12.39%.

2-(4-*Tert*-butylbenzoyl)-*N*-(4-methylphenyl)hydrazinecarbothioamide (**17**)

CAS Registry Number: 540498-64-4. Yield: 75%; M.p.: 162ºC. ^1^H NMR (DMSO-*d_6_*) δ (ppm) = 1.31 (s, 9H, 3xCH_3_), 2.28 (s, 3H, CH_3_), 7.11-7.14 (d, 2H, ArH, *J* = 9 Hz), 7.24-7.35 (m, 2H, ArH), 7.50-7.53 (d, 2H, ArH, *J* = 9 Hz), 7.89-7.91 (d, 2H, ArH, *J* = 9 Hz), 9.65 (s, 1H, NH), 9.73 (s, 1H, NH), 10.46 (s, 1H, NH); ^13^C NMR (DMSO-*d_6_*) δ (ppm) = 21.1 (CH_3_), 31.4 (3xCH_3_), 34.1 (C_t-butyl_), 120.9, 125.9, 130.3, 130.8, 132.1, 132.8, 137.7, 152.9 (12C_ar_), 159.9 (C=O), 166.8 (C=S); Analysis for C_19_H_23_N_3_OS (341.47) Calculated: C: 66.83%, H: 6.79%, N: 12.31%; Found: C: 66.91%, H: 6.70%, N: 12.27%.

2-(4-*Tert*-butylbenzoyl)-*N*-(4-methoxyphenyl)hydrazinecarbothioamide (**18**)

CAS Registry Number: 444075-82-5. Yield: 54%; M.p.: 110ºC. ^1^H NMR (DMSO-*d_6_*) δ (ppm) = 1.31 (s, 9H, 3xCH_3_), 3.74 (s, 3H, CH_3_), 6.87-6.90 (d, 2H, ArH, *J* = 9 Hz), 7.27-7.29 (d, 2H, ArH, *J* = 6 Hz), 7.50-7.53 (d, 2H, ArH, *J* = 9 Hz), 7.88-7.91 (d, 2H, ArH, *J* = 9 Hz), 9.61 (s, 1H, NH), 9.68 (s, 1H, NH), 10.45 (s, 1H, NH); ^13^C NMR (DMSO-*d_6_*) δ (ppm): 31.4 (3xCH_3_), 35.2 (C_t-butyl_), 56.1 (CH_3_), 113.6, 122.2, 125.4, 128.2, 130.3, 132.6, 133.6, 155.2 (12C_ar_), 157.1 (C=O), 166.2 (C=S); Analysis for C_19_H_23_N_3_O_2_S (357.47) Calculated: C: 63.84%, H: 6.49%, N: 11.75%; Found: C: 63.94%, H: 6.52%, N: 11.79%.

Detailed physicochemical data of 4,5-disubstituted 1,2,4-triazole-3-thione derivatives (**19**-**34**)

5-(3-Methoxyphenyl)-4-methyl-2,4-dihydro-3*H*-1,2,4-triazole-3-thione (**19**)

CAS Registry Number: 348593-44-2. Yield: 96%; M.p.: 128-132ºC. ^1^H NMR (DMSO-*d_6_*) δ (ppm) = 3.30 (s, 3H, CH_3_), 3.81 (s, 3H, CH_3_), 7.13-7.17 (m, 1H, ArH), 7.24-7.28 (m, 2H, ArH), 7.44-7.50 (t, 1H, ArH, *J* = 9 Hz), 13.90 (s, 1H, NH); ^13^C NMR (DMSO-*d_6_*) δ (ppm) = 33.1 (CH_3_), 56.0 (OCH_3_), 112.7, 114.6, 119.9, 128.3, 131.5,147.4 (6C­_ar_), 160.4 (C_triazole_), 169.3 (C=S); Analysis for C_10_H_11_N_3_OS (221.28) Calculated: C: 54.28%, H: 5.01%, N: 18.99%; Found: C: 54.36%, H: 5.07%, N: 18.90%.

4-Ethyl-5-(3-methoxyphenyl)-2,4-dihydro-3*H*-1,2,4-triazole-3-thione (**20**)

CAS Registry Number: 727717-92-2. Yield: 70%; M.p.: 106-108ºC. ^1^H NMR (DMSO-*d_6_*) δ (ppm) = 1.10-1.16 (t, 3H, CH_3_, *J* = 9 Hz), 3.98-4.07 (q, 2H, CH_2_, *J* = 9 Hz), 7.14-7.23 (m, 3H, ArH), 7.45-7.51 (t, 1H, ArH, *J* = 9 Hz), 13.89 (s, 1H, NH); ^13^C NMR (DMSO-*d_6_*) δ (ppm) = 14.5 (CH_3_), 45.2 (CH_2_), 56.1 (OCH_3_), 114.8, 119.4, 122.3, 128.2, 131.2, 147.2 (6C­_ar_), 160.4 (C_triazole_), 169.2 (C=S); Analysis for C_11_H_13_N_3_OS (235.31) Calculated: C: 56.15%, H: 5.57%, N: 17.86%; Found: C: 56.03%, H: 5.62%, N: 17.93%.

5-(3-Methoxyphenyl)-4-propyl-2,4-dihydro-3*H*-1,2,4-triazole-3-thione (**21**)

CAS Registry Number: 1546972-14-8. Yield: 53%; M.p.: 58-60ºC. ^1^H NMR (DMSO-*d_6_*) δ (ppm) = 0.67-0.73 (t, 3H, CH_3_, *J* = 9 Hz), 1.46-1.61 (m, 2H, CH_2_), 3.80 (s, 3H, CH_3_), 3.96-4.02 (t, 2H, CH_2_, *J* = 9 Hz), 7.13-7.22 (m, 3H, ArH), 7.44-7.51 (t, 1H, ArH, *J* = 12 Hz, *J* = 9 Hz), 13.90 (s, 1H, NH); ^13^C NMR (DMSO-*d_6_*) δ (ppm) = 12.0 (CH_3_), 21.7 (CH_2_), 49.0 (CH_2_), 56.2 (OCH_3_), 112.4, 114.8, 119.4, 128.2, 131.2, 148.3 (6C_ar_), 160.5 (C_triazole_), 169.6 (C=S); Analysis for C_12_H_15_N_3_OS (249.33) Calculated: C: 57.81%, H: 6.06%, N: 16.85%; Found: C: 57.89%, H: 6.11%, N: 16.78%.

4-Butyl-5-(3-methoxyphenyl)-2,4-dihydro-3*H*-1,2,4-triazole-3-thione (**22**)

Yield: 29%; M.p.: 78-82ºC. ^1^H NMR (DMSO-*d_6_*) δ (ppm) = 0.72-0.77 (t, 3H, CH_3_, *J* = 6 Hz, *J* = 9 Hz), 1.07-1.19 (m, 2H, CH_2_), 1.45-1.55 (m, 2H, CH_2_), 3.82 (s, 3H, CH_3_), 4.01-4.06 (t, 2H, CH_2_, *J* = 9 Hz, *J* = 6 Hz), 7.15-7.19 (m, 1H, ArH), 7.21-7.24 (m, 1H, ArH), 7.46-7.52 (t, 2H, CH_2_, *J* = 9 Hz), 13.92 (s, 1H, NH); ^13^C NMR (DMSO-*d_6_*) δ (ppm) = 14.0 (CH_3_), 20.5 (CH_2_), 30.1 (CH_2_), 46.5 (CH_2_), 56.1 (OCH_3_), 112.4, 114.8, 119.4, 128.2, 131.2, 148.3 (6C_ar_), 160.1 (C_triazole_), 169.6 (C=S); Analysis for C_13_H_17_N_3_OS (263.36) Calculated: C: 59.29%, H: 6.51%, N: 15.96%; Found: C: 59.34%, H: 6.47%, N: 16.02%.

4-Cyclohexyl-5-(3-methoxyphenyl)-2,4-dihydro-3*H*-1,2,4-triazole-3-thione (**23**)

Yield: 46%; M.p.: 162-164ºC. ^1^H NMR (DMSO-*d_6_*) δ (ppm) = 0.91-1.00 (m, 1H, CH_2-cyclohexyl_), 1.09-1.22 (m, 2H, CH_2-cyclohexyl_), 1.51-1.55 (m, 1H, CH_2-cyclohexyl_), 1.70-1.74 (m, 4H, CH_2-cyclohexyl_), 2.14-2.17 (m, 2H, CH_2-cyclohexyl_), 3.81 (s, 3H, CH_3_), 4.21-4.29 (m, 1H, CH _cyclohexyl_), 7.09-7.12 (m, 2H, ArH), 7.15-7.19 (m, 1H, ArH), 7.45-7.50 (t, 1H, ArH, *J* = 6 Hz, *J* = 9 Hz), 13.88 (s, 1H, NH), ^13^C NMR (DMSO-*d_6_*) δ (ppm) = 25.1 (2xCH_2-cyclohexyl_), 25.9 (2xCH_2-cyclohexyl_), 29.8 (CH_2-cyclohexyl_), 55.8 (CH_cyclohexyl­­­_), 57.5 (OCH_3_), 115.7, 116.9, 122.3, 128.5, 130.4, 151.8 (6C_ar_), 159.6 (C_triazole_), 166.6 (C=S); Analysis for C_15_H_19_N_3_OS (289.40) Calculated: C: 62.25%, H: 6.62%, N: 14.52%; Found: C: 62.32%, H: 6.69%, N: 14.59%.

4-Benzyl-5-(3-methoxyphenyl)-2,4-dihydro-3*H*-1,2,4-triazole-3-thione (**24**)

Yield: 88%; M.p.: 162-164ºC. ^1^H NMR (DMSO-*d_6_*) δ (ppm) = 3.52 (s, 3H, CH_3_), 5.34 (s, 2H, CH_2_), 6.99-7.10 (m, 5H, ArH), 7.23-7.31 (m, 3H, ArH), 7.35-7.40 (t, 1H, ArH, *J* = 6 Hz, *J* = 9 Hz), 14.15 (s, 1H, NH); ^13^C NMR (DMSO-*d_6_*) δ (ppm) = 47.3 (CH_2_), 55.6 (OCH_3_), 113.8, 117.3, 121.1, 126.9, 127.6, 127.9, 129.1, 130.6, 136.3, 151.8 (12C_ar_), 159.7 (C_triazole_), 168.5 (C=S); Analysis for C_16_H_15_N_3_OS (297.37) Calculated: C: 64.62%, H: 5.08%, N: 14.13%; Found: C: 64.69%, H: 5.02%, N: 14.20%.

5-(3-Methoxyphenyl)-4-(4-methylphenyl)-2,4-dihydro-3*H*-1,2,4-triazole-3-thione (**25**)

CAS Registry Number: 727717-75-1. Yield: 96%; M.p.: 202ºC. ^1^H NMR (DMSO-*d_6_*) δ (ppm) = 3.29 (s, 3H, CH_3_), 3.82 (s, 3H, CH_3_), 7.12-7.17 (m, 3H, ArH), 7.29-7.31 (m, 2H, ArH), 7.39-7.44 (t, 1H, ArH, *J* = 9 Hz, *J* = 6 Hz), 7.52-7.55 (d, 2H, ArH, *J* = 9 Hz), 13.75 (s, 1H, NH); ^13^C NMR (DMSO-*d_6_*) δ (ppm) = 21.0 (CH_3_), 55.8 (OCH­_3_), 113.5, 118.1, 120.6, 126.5, 128.9, 129.9, 134.4, 134.7, 137.1, 159.5 (12C_ar_), 160.1 (C_triazole_), 169.1 (C=S); Analysis for C_16_H_15_N_3_OS (297.37) Calculated: C: 64.62%, H: 5.08%, N: 14.13%; Found: C: 64.71%, H: 5.02%, N: 14.19%.

5-(3-Methoxyphenyl)-4-(4-methoxyphenyl)-2,4-dihydro-3*H*-1,2,4-triazole-3-thione (**26**)

CAS Registry Number: 730976-57-5. Yield: 97%; M.p.: 184-186ºC. ^1^H NMR (DMSO-*d_6_*) δ (ppm) = 3.62 (s, 3H, CH_3_), 3.80 (s, 3H, CH_3_), 6.84-6.86 (m, 1H, ArH), 6.90-6.93 (d, 1H, ArH, *J* = 9 Hz), 6.97-7.00 (d, 1H, ArH, *J* = 9 Hz), 7.02 (s, 1H, ArH), 7.05 (s, 1H, ArH), 7.25-7.30 (m, 3H, ArH), 14.10 (s, 1H, NH); ^13^C NMR (DMSO-*d_6_*) δ (ppm) = 56.1 (CH_3_), 56.4 (OCH_3_), 114.0, 114.9, 116.5, 120.9, 127.5, 127.6, 130.3, 130.4, 150.9, 159.3 (12C_ar_), 160.0, (C_triazol­e_), 169.3 (C=S); Analysis for C_16_H_15_N_3_O_2_S (313.37) Calculated: C: 61.32%, H: 4.82%, N: 13.41%; Found: C: 61.39%, H: 4.77%, N: 13.49%.

5-(4-*Tert*-butylphenyl)-4-methyl-2,4-dihydro-3*H*-1,2,4-triazole-3-thione (**27**)

CAS Registry Number: 175276-75-2. Yield: 62%; M.p.: 204-206ºC. ^1^H NMR (DMSO-*d_6_*) δ (ppm) = 1.31 (s, 9H, 3xCH_3_), 3.52 (s, 3H, CH_3_), 7.55-7.58 (d, 2H, ArH, *J* = 9 Hz), 7.63-7.67 (d, 2H, ArH, *J* = 12 Hz), 13.88 (s, 1H, NH); ^13^C NMR (DMSO-*d_6_*) δ (ppm) = 31.4 (3xCH_3_), 33.1 (CH_3_), 34.3 (C_t-butyl_), 126.2, 127.6, 130.1, 148.3 (6C_ar_), 151.3 (C_triazole_), 169.9 (C=S); Analysis for C_13_H_17_N_3_S (247.36) Calculated: C: 63.12%, H: 6.93%, N: 16.99%; Found: C: 63.21%, H: 6.88%, N: 17.07%.

5-(4-*Tert*-butylphenyl)-4-ethyl-2,4-dihydro-3*H*-1,2,4-triazole-3-thione (**28**)

Yield: 79%; M.p.: 158-160ºC. ^1^H NMR (DMSO-*d_6_*) δ (ppm) = 1.15-1.20 (t, 3H, CH_3_, *J* = 9 Hz, *J* = 6 Hz), 1.32 (s, 9H, 3xCH_3_), 3.99-4.07 (q, 2H, CH_­2_, *J* = 9 Hz), 7.57-7.63 (m, 4H, ArH), 13.92 (s, 1H, NH); ^13^C NMR (DMSO-*d_6_*) δ (ppm) = 13.9 (CH_3_), 31.4 (3xCH_3_), 35.1 (C_t-butyl_), 45.2 (CH_2_), 123.8, 126.4, 128.7, 151.6 (6C_ar_), 153.9 (C_triazole_), 167.2 (C=S); Analysis for C_14_H_19_N_3_S (261.39) Calculated: C: 64.33%, H: 7.33%, N: 16.08%; Found: C: 64.41%, H: 7.28%, N: 16.14%.

5-(4-*Tert*-butylphenyl)-4-propyl-2,4-dihydro-3*H*-1,2,4-triazole-3-thione (**29**)

Yield: 84%; M.p.: 172-176ºC. ^1^H NMR (DMSO-*d_6_*) δ (ppm) = 0.70-0.75 (t, 3H, CH_3_, *J* = 9 Hz, *J* = 6 Hz), 1.38 (s, 9H, 3xCH_3_), 1.52-1.64 (m, 2H, CH_2_), 3.97-4.02 (t, 2H, CH_2_, *J* = 9 Hz, *J* = 6 Hz), 7.57-7.64 (m, 4H, ArH), 13.91 (s, 1H, NH); ^13^C NMR (DMSO-*d_6_*) δ (ppm) = 11.9 (CH_3_), 21.7 (CH_2_), 31.4 (3xCH_3_), 34.3 (C_t-butyl_), 49.1 (CH_2_), 125.8, 127.6, 130.5, 148.8 (6C_ar_), 150.7 (C_triazole_), 169.7 (C=S); Analysis for C_15_H_21_N_3_S (275.41) Calculated: C: 65.41%, H: 7.69%, N: 15.26%; Found: C: 65.49%, H: 7.61%, N: 15.33%.

4-Butyl-5-(4-*tert*-butylphenyl)-2,4-dihydro-3*H*-1,2,4-triazole-3-thione (**30**)

CAS Registry Number: 522628-77-9. Yield: 79%; M.p.: 140-142ºC. ^1^H NMR (DMSO-*d_6_*) δ (ppm) = 0.71-0.77 (t, 3H, CH_3_, *J* = 9 Hz), 1.06-1.21 (m, 2H, CH_2_), 1.47-1.59 (m, 2H, CH_2_), 3.99-4.05 (t, 2H, CH_2_, *J* = 9 Hz), 7.55-7.88 (m, 4H, ArH), 13.86 (s, 1H, NH); ^13^C NMR (DMSO-*d_6_*) δ (ppm) = 13.7 (CH_3_), 19.5 (CH_2_), 30.0 (CH_2_), 31.4 (3xCH­_3_), 35.1 (C_t-butyl_), 43.9 (CH_2_), 123.8, 125.8, 126.3, 128.7, 129.6, 151.6 (6C_ar_), 153.9 (C_triazole_), 167.4 (C=S); Analysis for C_16_H_23_N_3_S (289.44) Calculated: C: 66.39%, H: 8.01%, N: 14.52%; Found: C: 66.44%, H: 8.09%, N: 14.59%.

5-(4-*Tert*-butylphenyl)-4-cyclohexyl-2,4-dihydro-3*H*-1,2,4-triazole-3-thione (**31**)

CAS Registry Number: 309272-76-2. Yield: 58%; M.p.: 240-242ºC. ^1^H NMR (DMSO-*d_6_*) δ (ppm) = 0.92-1.00 (m, 1H, CH_2-cyclohexyl_), 1.12-1.22 (m, 2H, CH_2-cyclohexyl_), 1.33 (s, 9H, 3xCH_3_), 1.53-1.56 (m, 1H, CH_2-cyclohexyl_), 1.70-1.73 (m, 4H, CH_2-cyclohexyl_), 2.25-2.29 (m, 2H, CH_2-cyclohexyl_), 4.20-4.26 (m, 1H, CH_cyclohexyl­_), 7.46-7.48 (d, 2H, ArH, *J* = 6 Hz), 7.57-7.59 (d, 2H, ArH, *J* = 6 Hz), 13.86 (s, 1H, NH); ^13^C NMR (DMSO-*d_6_*) δ (ppm) = 25.1 (2xCH­_2-cyclohexyl_), 25.8 (2xCH­_2-cyclohexyl_), 29.6 (CH_2-cyclohexyl_), 31.4 (3xCH_3_), 35.2 (C_t-butyl­_), 57.3 (CH_cyclohexyl­_), 124.4, 126.0, 129.9, 152.0 (6C_ar_), 153.9 (C_triazole_), 166.5 (C=S); Analysis for C_18_H_25_N_3_S (315.48) Calculated: C: 68.53%, H: 7.99%, N: 13.32%; Found: C: 68.62%, H: 7.90%, N: 13.39%.

4-Benzyl-5-(4-*tert*-butylphenyl)-2,4-dihydro-3*H*-1,2,4-triazole-3-thione (**32**)

Yield: 92%; M.p.: 190-192ºC. ^1^H NMR (DMSO-*d_6_*) δ (ppm) = 1.27 (s, 9H, 3xCH_3_), 5.34 (s, 2H, CH_2_), 7.02-7.04 (d, 2H, ArH, *J* = 6 Hz), 7.24-7.31 (m, 3H, ArH), 7.46-7.48 (d, 4H, ArH, *J* = 6 Hz), 14.11 (s, 1H, NH); ^13^C NMR (DMSO-*d_6_*) δ (ppm) = 31.3 (3xCH_3_), 35.1 (C_t-butyl_), 47.3 (CH_2_), 123.6, 126.2, 126.8, 127.9, 128.5, 129.1, 136.3, 151.9 (12C_ar_), 154.0 (C_triazole_), 168.5 (C=S); Analysis for C_19_H_21_N_3_S (323.46) Calculated: C: 70.55%, H: 6.54%, N: 12.99%; Found: C: 70.63%, H: 6.58%, N: 13.07%.

5-(4-*Tert*-butylphenyl)-4-(4-methylphenyl)-2,4-dihydro-3*H*-1,2,4-triazole-3-thione (**33**)

CAS Registry Number: 727704-60-1. Yield: 97%; M.p.: 242ºC. ^1^H NMR (DMSO-*d_6_*) δ (ppm) = 1.22 (s, 9H, 3xCH_3_), 2.37 (s, 3H, CH_3_), 7.22-7.26 (m, 4H, ArH), 7.30-7.33 (m, 2H, ArH), 7.35-7.48 (m, 2H, ArH), 14.09 (s, 1H, NH); ^13^C NMR (DMSO-*d_6_*) δ (ppm) = 21.1 (CH_3_), 31.4 (3xCH_3_), 34.3 (C_t-butyl­_), 125.3, 127.5, 130.5, 131.1, 131.3, 136.5, 138.9, 149.6 (12C_ar_), 151.1 (C_triazole_), 168.2 (C=S); Analysis for C_19_H_21_N_3_S (323.46) Calculated: C: 70.55%, H: 6.54%, N: 12.99%; Found: C: 70.61%, H: 6.50%, N: 13.09%.

5-(4-*Tert*-butylphenyl)-4-(4-methoxyphenyl)-2,4-dihydro-3*H*-1,2,4-triazole-3-thione (**34**)

CAS Registry Number: 713098-07-8. Yield: 59%; M.p.: 150-154ºC. ^1^H NMR (DMSO-*d_6_*) δ (ppm) = 1.31 (s, 9H, 3xCH_3_), 3.81 (s, 3H, CH_3_), 6.83-6.86 (d, 1H, ArH, *J* = 9 Hz), 7.03-7.06 (d, 1H, ArH, *J* = 9 Hz), 7.21-7.30 (m, 2H, ArH), 7.35-7.39 (m, 2H, ArH), 7.50-7.53 (d, 1H, ArH, *J* = 9 Hz), 7.85-7.89 (m, 1H, ArH), 14.06 (s, 1H, NH); ^13^C NMR (DMSO-*d_6_*) δ (ppm) = 31.3 (3xCH_3_), 35.1 (C_t-butyl_), 55.9 (CH_3_), 114.3, 125.6, 125.8, 127.5, 128.2, 129.7, 145.8, 154.8 (12C_ar_), 160.0 (C_triazole_), 169.3 (C=S); Analysis for C_19_H_21_N_3_OS (339.45) Calculated: C: 67.23%, H: 6.24%, N: 12.38%; Found: C: 67.32%, H: 6.29%, N: 12.46%.

Detailed physicochemical data of new pipemidic acid derivatives (**35**-**50**)

8-Ethyl-2-{4-[(3-(3-methoxyphenyl)-4-methyl-5-thioxo-4,5-dihydro-1*H*-1,2,4-triazol-1-yl)methyl]piperazin-1-yl}-5-oxo-5,8-dihydropyrido[2,3-*d*]pyrimidine-6-carboxylic acid (**35**)

Yield: 96%; M.p.: 258-260ºC. ^1^H NMR (DMSO-*d_6_*) δ (ppm) = 1.30-1.35 (t, 3H, CH_3_, *J* = 6 Hz, *J* = 9 Hz), 2.83-2.87 (m, 4H, 2xCH_2-piperazine­_), 3.54 (s, 3H, CH_3_), 3.79 (s, 3H, CH_3_), 3.92-3.96 (m, 4H, 2xCH_2-piperazine_), 4.32-4.40 (q, 2H, CH_2_, *J* = 9 Hz, *J* = 6 Hz), 5.20 (s, 2H, CH_2_), 7.13-7.16 (m, 1H, ArH), 7.24-7.27 (m, 2H, ArH), 7.43-7.50 (t, 1H, ArH, *J* = 9 Hz, *J* = 12 Hz), 8.93 (s, 1H, =CH), 9.17 (s, 1H, OH), 9.18 (s, 1H, ArH); ^13^C NMR (DMSO-*d_6_*) δ (ppm) = 12.6 (CH_3_), 33.1 (CH_3_), 45.4 (CH_2_), 46.5 (2xCH_2-piperazine_), 51.4 (2xCH_2-piperazine_), 56.0 (OCH_3_), 63.5 (CH_2_), 106.3, 112.9, 113.5, 115.0, 120.1, 128.5, 131.6, 150.1, 155.6, 159.6 (10C_ar_), 160.5 (C_triazole_), 160.5 (C_ar_), 167.1 (C=O), 169.8 (C_ar_), 172.2 (C=S), 177.2 (C=O); Analysis for C_25_H_28_N_8_O_4_S (536.61) Calculated: C: 55.96%, H: 5.26%, N: 20.88%; Found: C: 56.03%, H: 5.21%, N: 20.93%.

8-Ethyl-2-{4-[(3-(3-methoxyphenyl)-4-ethyl-5-thioxo-4,5-dihydro-1*H*-1,2,4-triazol-1-yl)methyl]piperazin-1-yl}-5-oxo-5,8-dihydropyrido[2,3-*d*]pyrimidine-6-carboxylic acid (**36**)

Yield: 98%; M.p.: 180-182ºC. ^1^H NMR (DMSO-*d_6_*) δ (ppm) = 1.10-1.16 (t, 3H, CH_3_, *J* = 9 Hz), 1.30-1.35 (t, 3H, CH_3_, *J* = 6 Hz, *J* = 9 Hz), 2.82-2.89 (m, 4H, 2xCH_2-piperazine_), 3.81 (s, 3H, CH_3_), 3.89-3.92 (m, 4H, 2xCH_2-piperazine_), 3.98-4.07 (q, 2H, CH_2_, *J* = 9 Hz), 4.33-4.40 (q, 2H, CH_2_, *J* = 9 Hz, *J* = 6 Hz), 5.19 (s, 2H, CH_2_), 7.14-7.23 (m, 3H, ArH), 7.45-7.51 (d, 1H, ArH, *J* = 9 Hz), 8.93 (s, 1H, =CH), 9.17 (s, 1H, OH), 9.19 (s, 1H, ArH); ^13^C NMR (DMSO-*d_6_*) δ (ppm) = 12.1 (CH_3_), 14.6 (CH_3_), 45.4 (CH_2_), 45.9 (CH_2_), 46.6 (2xCH_2-piperazine_), 51.1 (2xCH_2-piperazine_), 56.1 (OCH_3_), 63.6 (CH_2_), 106.2, 112.6, 113.6, 115.1, 119.7, 128.4, 131.3, 150.1, 155.6, 159.6, 160.6 (11C_ar_), 164.2 (C_triazole_), 167.1 (C=O), 169.8 (C_ar_), 172.7 (C=S), 177.1 (C=O); Analysis for C_26_H_30_N_8_O_4_S (550.63) Calculated: C: 56.71%, H: 5.49%, N: 20.35%; Found: C: 56.77%, H: 5.42%, N: 20.44%.

8-Ethyl-2-{4-[(3-(3-methoxyphenyl)-4-propyl-5-thioxo-4,5-dihydro-1*H*-1,2,4-triazol-1-yl)methyl]piperazin-1-yl}-5-oxo-5,8-dihydropyrido[2,3-*d*]pyrimidine-6-carboxylic acid (**37**)

Yield: 98%; M.p.: 178-180ºC. ^1^H NMR (DMSO-*d_6_*) δ (ppm) = 0.67-0.73 (t, 3H, CH_3_, *J* = 9 Hz), 1.32-1.37 (t, 3H, CH_3_, *J* = 9 Hz, *J* = 6 Hz), 1.49-1.58 (m, 2H, CH_2_), 2.82-2.86 (m, 4H, 2xCH_2-piperazine_), 3.80 (s, 3H, CH_3_), 3.86-3.91 (m, 4H, 2xCH_2-piperazine_), 3.96-4.01 (t, 2H, CH_2_, *J* = 9 Hz, *J* = 6 Hz), 4.34-4.40 (q, 2H, CH_2_, *J* = 6 Hz, *J* = 9 Hz), 5.20 (s, 2H, CH_2_), 7.13-7.22 (m, 3H, ArH), 7.44-7.51 (t, 1H, ArH, *J* = 12 Hz, *J* = 9 Hz), 8.94 (s, 1H, =CH), 9.18 (s, 1H, OH), 9.19 (s, 1H, ArH); ^13^C NMR (DMSO-*d_6_*) δ (ppm) = 11.9 (CH_3_), 12.4 (CH_3_), 45.5 (CH_2_), 46.3 (2xCH_2-piperazine_), 49.9 (CH_2_), 51.1 (2xCH_2-piperazine_), 56.1 (OCH_3_), 63.5 (CH_2_), 106.3, 112.6, 113.5, 115.1, 119.7, 128.4, 131.3, 150.1, 155.5, 159.3, 160.6 (11C_ar_), 163.8 (C_triazole_), 167.1 (C=O), 169.7 (C_ar_), 173.5 (C=S), 177.2 (C=O); Analysis for C_27_H_32_N_8_O_4_S (564.66) Calculated: C: 57.43%, H: 5.71%, N: 19.84%; Found: C: 57.51%, H: 5.67%, N: 19.90%.

8-Ethyl-2-{4-[(4-butyl-3-(3-methoxyphenyl)-5-thioxo-4,5-dihydro-1*H*-1,2,4-triazol-1-yl)methyl]piperazin-1-yl}-5-oxo-5,8-dihydropyrido[2,3-*d*]pyrimidine-6-carboxylic acid (**38**)

Yield: 98%; M.p.: 146-148ºC. ^1^H NMR (DMSO-*d_6_*) δ (ppm) = 0.71-0.76 (t, 3H, CH_3_, *J* = 9 Hz, *J* = 6 Hz), 1.05-1.16 (m, 2H, CH_2_), 1.31-1.37 (t, 3H, CH_3_, *J* = 12 Hz, *J* = 6 Hz), 1.44-1.54 (m, 2H, CH_2_), 2.83-2.89 (m, 4H, 2xCH_2-piperazine_), 3.80 (s, 3H, CH_3_), 3.83-3.93 (m, 4H, 2xCH_2- piperazine_), 4.00-4.05 (t, 2H, CH_2_, *J* = 6 Hz, *J* = 9 Hz), 4.33-4.41 (q, 2H, CH_2_, *J* = 9 Hz), 5.20 (s, 2H, CH_2_), 7.13-7.23 (m, 3H, ArH), 7.44-7.50 (t, 1H, ArH, *J* = 9 Hz, *J* = 6 Hz), 8.93 (s, 1H, =CH), 9.18 (s, 1H, OH), 9.19 (s, 1H, ArH); ^13^C NMR (DMSO-*d_6_*) δ (ppm) = 12.4 (CH_3_), 14.1 (CH_3_), 20.5 (CH_2_), 31.1 (CH_2_), 45.5 (CH_2_), 46.3 (2xCH_2-piperazine_), 47.3 (CH_2_), 51.1 (2xCH_2-piperazine­­_), 56.1 (OCH_3_), 63.3 (CH_2_), 106.6, 112.1, 113.3, 115.1, 119.7, 128.4, 131.3, 150.1, 155.6 159.8, 160.6 (11C_ar_), 163.8 (C_triazole_), 167.1 (C=O), 169.8 (C_ar_), 173.3 (C=S), 177.1 (C=O); Analysis for C_28_H_34_N_8_O_4_S (578.69) Calculated: C: 58.11%, H: 5.92%, N: 19.36%; Found: C: 58.20%, H: 5.89%, N: 19.42%.

8-Ethyl-2-{4-[(4-cyclohexyl-3-(3-methoxyphenyl)-5-thioxo-4,5-dihydro-1*H*-1,2,4-triazol-1-yl)methyl]piperazin-1-yl}-5-oxo-5,8-dihydropyrido[2,3-*d*]pyrimidine-6-carboxylic acid (**39**)

Yield: 97%; M.p.: 196-200ºC. ^1^H NMR (DMSO-*d_6_*) δ (ppm) = 0.88-1.04 (m, 1H, CH_2-cyclohexyl_), 1.06-1.20 (m, 2H, CH_2-cyclohexyl_), 1.29-1.34 (t, 3H, CH_3_, *J* = 9 Hz, *J* = 6 Hz), 1.49-1.54 (m, 1H, CH_2-cyclohexyl_), 1.67-1.75 (m, 4H, 2xCH_2-cyclohexyl_), 2.05-2.13 (m, 2H, CH_2- cyclohexyl_), 2.70-2.76 (q, 1H, CH, *J* = 6 Hz), 2.78-2.85 (m, 4H, 2xCH_2-piperazine_), 3.77 (s, 3H, CH_3_), 3.88-3.95 (m, 4H, CH_2-piperazine_), 4.23-4.28 (m, 1H, CH_cyclohexyl_), 4.32-4.40 (q, 2H, CH_2_, *J* = 9 Hz, *J* = 6 Hz), 5.18 (s, 2H, CH_2_), 7.08-7.18 (m, 3H, ArH), 7.42-7.48 (m, 1H, ArH), 8.94-8.95 (d, 1H, =CH, *J* = 3 Hz), 9.16 (s, 1H, OH), 9.18 (s, 1H, ArH); ^13^C NMR (DMSO-*d_6_*) δ (ppm) = 12.4 (CH_3_), 24.7 (2xCH_2-cyclohexyl­_), 25.9 (CH_2-cyclohexyl_), 31.5 (2xCH_2-cyclohexyl_), 45.8 (CH_2_), 46.8 (2xCH_2-piperazine_), 51.3 (2xCH_2-piprazine_), 56.1 (OCH_3_), 56.3 (CH_cyclohexyl­_), 63.5 (CH_2_), 106.5, 112.3, 113.3, 115.3, 119.2, 128.4, 131.1, 150.1, 155.6 (9C_ar_), 157.8 (C_triazole_), 159.6, 160.6 (2C_ar_), 167.0 (C=O), 169.8 (C_ar_), 175.6 (C=S), 177.3 (C=O); Analysis for C_30_H_36_N_8_O_4_S (604.72) Calculated: C: 59.58%, H: 6.00%, N: 18.53%; Found: C: 59.63%, H: 6.05%, N: 18.47%.

8-Ethyl-2-{4-[(4-benzyl-3-(3-methoxyphenyl)-5-thioxo-4,5-dihydro-1*H*-1,2,4-triazol-1-yl)methyl]piperazin-1-yl}-5-oxo-5,8-dihydropyrido[2,3-*d*]pyrimidine-6-carboxylic acid (**40**)

Yield: 96%; M.p.: 208-210ºC. ^1^H NMR (DMSO-*d_6_*) δ (ppm) = 1.32-1.37 (t, 3H, CH_3_, *J* = 9 Hz, *J* = 6 Hz), 2.76-2.81 (m, 4H, 2xCH­_2-piperazine_), 2.86-2.92 (q, 1H, CH, *J* = 6 Hz), 3.61 (s, 3H, CH_3_), 3.81-3.91 (m, 4H, 2xCH_2-piperazine­_), 4.33-4.41 (q, 2H, CH_2_, *J* = 9 Hz, *J* = 6 Hz), 5.27 (s, 2H, CH_2_), 5.35 (s, 2H, CH_2_), 6.97-7.08 (m, 5H, ArH), 7.22-7.25 (d, 2H, ArH, *J* = 9 Hz), 7.35-7.39 (t, 2H, ArH, *J* = 9 Hz, *J* = 12 Hz), 8.94-8.95 (d, 1H, =CH, *J* = 3 Hz), 9.19 (s, 1H, OH), 9.20 (s, 1H, ArH); ^13^C NMR (DMSO-*d_6_*) δ (ppm) = 12.5 (CH­_3_), 45.8 (CH_2_), 46.9 (2xCH_2-piperazine_), 51.4 (2xCH_2-piperazine_), 52.9 (CH_2_), 56.2 (OCH_3_), 63.4 (CH_2_), 106.6, 112.6, 113.5, 115.1, 119.7, 128.4, 128.5, 128.8, 131.3, 137.1, 150.8, 155.6 (14C_ar_), 157.8 (C_triazole_), 159.6, 160.7 (2C_ar_), 167.1 (C=O), 169.8 (C_ar_), 172.7 (C=S), 177.3 (C=O); Analysis for C_31_H_32_N_8_O_4_S (612.70) Calculated: C: 60.77%, H: 5.26%, N: 18.29%; Found: C: 60.81%, H: 5.22%, N: 18.36%.

8-Ethyl-2-{4-[(3-(3-methoxyphenyl)-4-(4-methylphenyl)-5-thioxo-4,5-dihydro-1*H*-1,2,4-triazol-1-yl)methyl]piperazin-1-yl}-5-oxo-5,8-dihydropyrido[2,3-*d*]pyrimidine-6-carboxylic acid (**41**)

Yield: 76%; M.p.: 214-218ºC. ^1^H NMR (DMSO-*d_6_*) δ (ppm) = 1.31-1.36 (t, 3H, CH_3_, *J* = 9 Hz, *J* = 6 Hz), 2.34 (s, 3H, CH_3_), 2.92-2.96 (m, 4H, 2xCH_2-piperazine_), 3.56 (s, 3H, CH_3_), 3.94-3.99 (m, 4H, 2xCH_2-piperazine_), 4.34-4.41 (q, 2H, CH_2_, *J* = 6 Hz), 5.27 (s, 2H, CH_2_), 6.77-6.79 (m, 2H, ArH), 6.91-6.98 (t, 2H, ArH, *J* = 9 Hz, *J* = 12 Hz), 7.21-7.30 (m, 4H, ArH), 8.94 (s, 1H, =CH), 9.18 (s, 1H, OH), 9.20 (s, 1H, ArH); ^13^C NMR (DMSO-*d_6_*) δ (ppm) = 12.6 (CH_3_), 21.1 (CH_3_), 45.8 (CH_2_), 46.6 (2xCH_2-piperazine_), 51.1 (2xCH_2-piperazine_), 56.0 (OCH_3_), 63.6 (CH_2_), 106.1, 112.1, 113.5, 115.5, 118.9, 128.4, 130.5, 130.9, 131.8, 136.2, 139.1, 150.1, 155.6 (15C_ar_), 157.9 (C_triazole_), 159.6, 160.7 (2C_ar_), 167.1 (C=O), 168.3 (C=S), 177.2 (C=O); Analysis for C_31_H_32_N_8_O_4_S (612.70) Calculated: C: 60.77%, H: 5.26%, N: 18.29%; Found: C: 60.82%, H: 5.22%, N: 18.33%.

8-Ethyl-2-{4-[(3-(3-methoxyphenyl)-4-(4-methoxyphenyl)-5-thioxo-4,5-dihydro-1*H*-1,2,4-triazol-1-yl)methyl]piperazin-1-yl}-5-oxo-5,8-dihydropyrido[2,3-*d*]pyrimidine-6-carboxylic acid (**42**)

Yield: 92%; M.p.: 216-220ºC. ^1^H NMR (DMSO-*d_6_*) δ (ppm) = 1.31-1.36 (t, 3H, CH_3_, *J* = 6 Hz, *J* = 9 Hz), 2.91-2.97 (m, 4H, 2xCH_2-piperazine_), 3.58 (s, 3H, CH_3_), 3.78 (s, 3H, CH_3_), 3.91-4.00 (m, 4H, 2xCH_2-piperazine_), 4.34-4.42 (q, 2H, CH_2_, *J* = 6 Hz, *J* = 9 Hz), 5.25 (s, 2H, CH_2_), 6.79-6.81 (m, 1H, ArH), 6.92-6.96 (m, 2H, ArH), 6.98-7.00 (t, 1H, ArH, *J* = 3 Hz), 7.02-7.04 (t, 1H, ArH, *J* = 3 Hz), 7.23-7.24 (d, 1H, ArH, *J* = 3 Hz), 7.27-7.30 (d, 2H, ArH, *J* = 9 Hz), 8.95 (s, 1H, =CH), 9.19 (s, 1H, OH), 9.21 (s, 1H, ArH); ^13^C NMR (DMSO-*d_6_*) δ (ppm) = 12.4 (CH_3_), 45.8 (CH_2_), 46.8 (2xCH_2-piperazine_), 51.4 (2xCH_2-piperazine_), 56.0 (2xOCH_3_), 63.5 (CH_2_), 106.5, 112.0, 113.5, 115.3, 115.4, 118.9, 128.4, 129.6, 130.5, 130.9, 150.0, 155.6 (14C_ar_), 157.9 (C_triazole_), 159.3, 159.6, 160.7 (3C_ar_), 167.0 (C=O), 168.3 (C=S), 169.8 (C_ar_), 177.2 (C=O); Analysis for C_31_H_32_N_8_O_5_S (628.70) Calculated: C: 59.22%, H: 5.13%, N: 17.82%; Found: C: 59.29%, H: 5.17%, N: 17.90%.

8-Ethyl-2-{4-[(3-(4-*tert*-butylphenyl)-4-methyl-5-thioxo-4,5-dihydro-1*H*-1,2,4-triazol-1-yl)methyl]piperazin-1-yl}-5-oxo-5,8-dihydropyrido[2,3-*d*]pyrimidine-6-carboxylic acid (**43**)

Yield: 92%; M.p.: 260-262ºC. ^1^H NMR (DMSO-*d_6_*) δ (ppm) = 1.29 (s, 9H, 3xCH_3_), 1.32-1.37 (t, 3H, CH_3_, *J* = 9 Hz), 2.82-2.86 (m, 4H, 2xCH_2-piperazine_), 3.55 (s, 3H, CH_3_), 3.88-3.98 (m, 4H, 2xCH_2-piperazine_), 4.31-4.41 (q, 2H, CH_2_, *J* = 9 Hz, *J* = 12 Hz), 5.19 (s, 2H, CH_2_), 7.53-7.57 (d, 2H, ArH, *J* = 12 Hz), 7.63-7.66 (d, 2H, ArH, *J* = 9 Hz), 8.94 (s, 1H, =CH), 9.17 (s, 1H, OH), 9.19 (s, 1H, ArH); ^13^C NMR (DMSO-*d_6_*) δ (ppm) = 12.3 (CH_3_), 31.1 (3xCH_3_), 33.1 (CH_3_), 34.3 (C_t-butyl_), 45.8 (CH_2_), 46.3 (2xCH_2-piperazine_), 51.3 (2xCH_2-piperazine_), 63.3 (CH_2_), 106.1, 113.4, 126.6, 128.2, 129.9, 150.1, 151.7, 155.6, 159.7 (11C_ar_), 160.1 (C_triazole_), 167.0 (C=O), 169.8 (C_ar_), 172.2 (C=S), 177.1 (C=O); Analysis for C_28_H_34_N_8_O_3_S (562.69) Calculated: C: 59.77%, H: 6.09%, N: 19.91%; Found: C: 59.83%, H: 6.03%, N: 19.97%.

8-Ethyl-2-{4-[(3-(4-*tert*-butylphenyl)-4-ethyl-5-thioxo-4,5-dihydro-1*H*-1,2,4-triazol-1-yl)methyl]piperazin-1-yl}-5-oxo-5,8-dihydropyrido[2,3-*d*]pyrimidine-6-carboxylic acid (**44**)

Yield: 92%; M.p.: 226-230ºC. ^1^H NMR (DMSO-*d_6_*) δ (ppm) = 1.14-1.19 (t, 3H, CH_3_, *J* = 6 Hz, *J* = 9 Hz), 1.29 (s, 9H, 3xCH_3_), 1.31-1.37 (t, 3H, CH_3_, *J* = 9 Hz), 2.81-2.89 (m, 4H, 2xCH_2-piperazine_), 3.82-3.95 (m, 4H, 2xCH_2-piperazine_), 4.00-4.08 (q, 2H, CH_2_, *J* = 6 Hz), 4.32-4.40 (q, 2H, CH_2_, *J* = 9 Hz, *J* = 6 Hz), 5.19 (s, 2H, CH_2_), 7.50-7.66 (m, 4H, ArH), 8.94 (s, 1H, =CH), 9.18 (s, 1H, OH), 9.19 (s, 1H, ArH); ^13^C NMR (DMSO-*d_6_*) δ (ppm) = 12.4 (CH_3_), 14.5 (CH_3_), 31.4 (3xCH_3_), 34.3 (C_t-butyl­_), 45.3 (CH_2_), 45.7 (CH_2_), 46.5 (2xCH_2-piperazine_), 51.3 (2xCH_2-piperazine_), 63.3 (CH_2_), 106.2, 113.5, 126.1, 128.1, 130.3, 150.6, 151.1, 155.1, 159.8 (11C_ar­_), 163.9 (C_triazole_), 167.0 (C=O), 169.8 (C_ar_), 172.7 (C=S), 177.0 (C=O); Analysis for C_29_H_36_N_8_O_3_S (576.71) Calculated: C: 60.40%, H: 6.29%, N: 19.43%; Found: C: 60.47%, H: 6.32%, N: 19.49%.

8-Ethyl-2-{4-[(3-(4-*tert*-butylphenyl)-4-propyl-5-thioxo-4,5-dihydro-1*H*-1,2,4-triazol-1-yl)methyl]piperazin-1-yl}-5-oxo-5,8-dihydropyrido[2,3-*d*]pyrimidine-6-carboxylic acid (**45**)

Yield: 92%; M.p.: 206-208ºC. ^1^H NMR (DMSO-*d_6_*) δ (ppm) = 0.66-0.72 (t, 3H, CH_3_, *J* = 9 Hz), 1.29 (s, 9H, 3xCH_­3_), 1.31-1.37 (t, 3H, CH_3_, *J* = 9 Hz), 1.52-1.62 (m, 2H, CH_2_), 2.82-2.88 (m, 4H, 2xCH_2-piperazine_), 3.80-3.94 (m, 4H, 2xCH_2-piperazine_), 3.98-4.04 (t, 2H, CH_2_, *J* = 9 Hz), 4.30-4.39 (q, 2H, CH_2_, *J* = 9 Hz, *J* = 6 Hz), 5.22 (s, 2H, CH_2_), 7.53-7.61 (m, 4H, ArH), 8.93 (s, 1H, =CH), 9.14 (s, 1H, OH), 9.16 (s, 1H, ArH); ^13^C NMR (DMSO-*d_6_*) δ (ppm) = 12.0 (CH_3_), 12.2 (CH_3_), 21.7 (CH_2_), 31.4 (3xCH_3_), 34.3 (C_t-butyl_), 45.5 (CH_2_), 46.2 (2xCH_2-piperazine_), 49.9 (CH_2_), 51.3 (2xCH_2-piperazine_), 61.3 (CH_2_), 106.3, 113.5, 126.1, 128.1, 130.3, 150.4, 151.1, 155.5, 159.5 (11C_ar_), 163.5 (C_triazole_), 167.1 (C=O), 169.8 (C_ar_), 173.3 (C=S), 177.1 (C=O); Analysis for C_30_H_38_N_8_O_3_S (590.74) Calculated: C: 60.99%, H: 6.48%, N: 18.97%; Found: C: 60.76%, H: 6.44%, N: 19.03%.

8-Ethyl-2-{4-[(4-butyl-3-(4-*tert*-butylphenyl)-5-thioxo-4,5-dihydro-1*H*-1,2,4-triazol-1-yl)methyl]piperazin-1-yl}-5-oxo-5,8-dihydropyrido[2,3-*d*]pyrimidine-6-carboxylic acid (**46**)

Yield: 94%; M.p.: 158-160ºC. ^1^H NMR (DMSO-*d_6_*) δ (ppm) = 0.72-0.77 (t, 3H, CH_3_, *J* = 6 Hz, *J* = 9 Hz), 1.04-1.18 (m, 2H, CH_2_), 1.29 (s, 9H, 3xCH_3_), 1.31-1.35 (t, 3H, CH_3_, *J* = 6 Hz), 1.47-1.58 (m, 2H, CH_2_), 2.81-2.87 (m, 4H, 2xCH_2-piperazine_), 3.90-3.98 (m, 4H, 2xCH_2- piperazine_), 4.01-4.06 (t, 2H, CH_2_, *J* = 9 Hz, *J* = 6 Hz), 4.33-4.40 (q, 2H, CH_2_, *J* = 6 Hz, *J* = 9 Hz), 5.20 (s, 2H, CH_2_), 7.52-7.61 (m, 4H, ArH), 8.94 (s, 1H, =CH), 9.17 (s, 1H, OH), 9.18 (s, 1H, ArH); ^13^C NMR (DMSO-*d_6_*) δ (ppm) = 12.1 (CH­_3_), 14.0 (CH_3_), 20.5 (CH_2_), 30.1 (CH_2_), 31.4 (3xCH_3_), 34.3 (C_t-butyl_), 45.8 (CH_2_), 46.7 (2xCH_2-piperazine_), 47.3 (CH_2_), 51.5 (2xCH_2-piperazine_), 63.6 (CH_2_), 106.5, 113.3, 126.1, 128.1, 130.3, 150.1, 155.6, 159.6, 163.1 (11C_ar_), 167.1 (C=O), 169.8 (C_ar_), 173.2 (C=S), 177.1 (C=O); Analysis for C_31_H_40_N_8_O_3_S (604.77) Calculated: C: 61.57%, H: 6.67%, N: 18.53%; Found: C: 61.63%, H: 6.70%, N: 18.59%.

8-Ethyl-2-{4-[(3-(4-*tert*-butylphenyl)-4-cyclohexyl-5-thioxo-4,5-dihydro-1*H*-1,2,4-triazol-1-yl)methyl]piperazin-1-yl}-5-oxo-5,8-dihydropyrido[2,3-*d*]pyrimidine-6-carboxylic acid (**47**)

Yield: 92%; M.p.: 218-220ºC. ^1^H NMR (DMSO-*d_6_*) δ (ppm) = 0.89-0.99 (m, 2H, CH_2-cyclohexyl_), 1.13-1.22 (m, 2H, CH_2-cyclohexyl_), 1.29 (s, 9H, 3xCH_3_), 1.30-1.35 (t, 3H, CH_3_, *J* = 9 Hz, *J* = 6 Hz), 1.50-1.55 (m, 2H, CH_2-cyclohexyl_), 1.68-1.73 (m, 4H, 2xCH_2-cyclohexyl­_), 2.82-2.86 (m, 4H, CH_2-piperazin­e_), 3.91-3.96 (m, 4H, 2xCH_2-piperazin­e_), 4.28-4.31 (m, 1H, CH_cyclohexyl_), 4.33-4.40 (q, 2H, CH_2_, *J* = 6 Hz), 5.18 (s, 2H, CH_2_), 7.44-7.47 (d, 2H, ArH, *J* = 9 Hz), 7.53-7.57 (d, 2H, ArH, *J* = 12 Hz), 8.94 (s, 1H, =CH), 9.18 (s, 1H, OH), 9.19 (s, 1H, ArH); ^13^C NMR (DMSO-*d_6_*) δ (ppm) = 12.5 (CH_3_), 24.8 (2xCH_2-cyclohexyl_), 25.9 (CH_2-cyclohexyl­_), 31.4 (3xCH_3_), 31.6 (2xCH_2-cyclohexyl­_), 34.4 (C_t-butyl_), 45.8 (CH_2_), 46.8 (2xCH_2-piperazine_), 51.5 (2xCH_2-piperazine_), 56.2 (CH_cyclohexyl_), 63.6 (CH_2_), 106.6, 113.6, 125.8, 128.2, 130.7, 150.1, 150.6, 155.6 (10C_ar_), 157.8 (C_triazole_), 159.6 (C_ar_), 167.1 (C=O), 169.8 (C_ar_), 175.6 (C=S), 177.4 (C=O); Analysis for C_33_H_42_N_8_O_3_S (630.81) Calculated: C: 62.83%, H: 6.71%, N: 17.76%; Found: C: 62.91%, H: 6.77%, N: 17.83%.

8-Ethyl-2-{4-[(4-benzyl-3-(4-*tert*-butylphenyl)-5-thioxo-4,5-dihydro-1*H*-1,2,4-triazol-1-yl)methyl]piperazin-1-yl}-5-oxo-5,8-dihydropyrido[2,3-*d*]pyrimidine-6-carboxylic acid (**48**)

Yield: 94%; M.p.: 222-224ºC. ^1^H NMR (DMSO-*d_6_*) δ (ppm) = 1.25 (s, 9H, 3xCH_3_), 1.32-1.36 (t, 3H, CH_3_, *J* = 6 Hz), 2.88-2.92 (m, 4H, CH_2-piperazine_), 3.94-4.00 (m, 4H, 2xCH_2-piperazine_), 4.35-4.42 (q, 2H, CH_2_, *J* = 6 Hz), 5.29 (s, 2H, CH_2_), 5.36 (s, 2H, CH_2_), 7.00-7.04 (m, 2H, ArH), 7.22-7.29 (m, 2H, ArH), 7.42-7.48 (m, 5H, ArH), 8.97 (s, 1H, =CH), 9.20 (s, 1H, OH), 9.21 (s, 1H, ArH); ^13^C NMR (DMSO-*d_6_*) δ (ppm) = 12.4 (CH_3_), 31.4 (3xCH_3_), 34.5 (C_t-butyl_), 45.8 (CH_2_), 46.9 (2xCH_2-piperazine_), 51.5 (2xCH_2-piperazine_), 52.8 (CH_2_), 63.6 (CH_2_), 106.6, 113.5, 128.0, 128.1, 128.5, 128.8, 130.3, 137.0, 150.0, 151.1, 155.6 (15C_ar_), 157.1 (C_triazole_), 159.6 (C_ar_), 167.0 (C=O), 169.8 (C_ar_), 172.7 (C=S), 177.2 (C=O); Analysis for C_34_H_38_N_8_O_3_S (638.78) Calculated: C: 63.93%, H: 6.00%, N: 17.54%; Found: C: 63.87%, H: 6.04%, N: 17.49%.

8-Ethyl-2-{4-[(3-(4-*tert*-butylphenyl)-4-(4-methylphenyl)-5-thioxo-4,5-dihydro-1*H*-1,2,4-triazol-1-yl)methyl]piperazin-1-yl}-5-oxo-5,8-dihydropyrido[2,3-*d*]pyrimidine-6-carboxylic acid (**49**)

Yield: 95%; M.p.: 230-232ºC. ^1^H NMR (DMSO-*d_6_*) δ (ppm) = 1.19 (s, 9H, 3xCH_3_), 1.31-1.36 (t, 3H, CH_3_, *J* = 6 Hz, *J* = 9 Hz), 2.35 (s, 3H, CH_3_), 2.92-2.95 (m, 4H, 2xCH_2-piperazine_), 3.95-3.98 (m, 4H, 2xCH_2-piperazine_), 4.33-4.41 (q, 2H, CH_2_, *J* = 9 Hz, *J* = 6 Hz), 5.26 (s, 2H, CH_2_), 7.22-7.25 (t, 5H, ArH, *J* = 9 Hz), 7.33-7.36 (d, 3H, ArH, *J* = 9 Hz), 8.93 (s, 1H, =CH), 9.18 (s, 1H, OH), 9,19 (s, 1H, ArH); ^13^C NMR (DMSO-*d_6_*) δ (ppm) = 12.3 (CH_3_), 21.1 (CH_3_), 31.4 (3xCH_3_), 34.3 (C_t-butyl_), 45.8 (CH_2_), 46.7 (2xCH_2-piperazine_), 51.4 (2xCH_2-piperazine_), 63.4 (CH_2_), 106.1, 113.6, 125.5,128.2, 130.5, 131.1, 131.8, 136.2, 139.1, 150.1, 155.1 (16C_ar_), 157.6 (C_triazole_), 159.7 (C_ar_), 167.1 (C=O), 168.3 (C=S), 169.8 (C_ar_), 177.1 (C=O); Analysis for C_34_H_38_N_8_O_3_S (638,78) Calculated: C: 63.93%, H: 6.00%, N: 17.54%; Found: C: 63.88%, H: 5.96%, N: 17.60%.

8-Ethyl-2-{4-[(3-(4-*tert*-butylphenyl)-4-(4-methoxyphenyl)-5-thioxo-4,5-dihydro-1*H*-1,2,4-triazol-1-yl)methyl]piperazin-1-yl}-5-oxo-5,8-dihydropyrido[2,3-*d*]pyrimidine-6-carboxylic acid (**50**)

Yield: 93%; M.p.: 242-246ºC. ^1^H NMR (DMSO-*d_6_*) δ (ppm) = 1.20 (s, 9H, 3xCH_3_), 1.31-1.37 (t, 3H, CH_3_, *J* = 9 Hz), 2.76-2.79 (m, 4H, 2xCH_2-piperazine_), 3.77 (s, 3H, CH_3_), 3.79-3.96 (m, 4H, 2xCH_2-piperazine_), 4.36-4.41 (q, 2H, CH_2_, *J* = 6 Hz), 5.26 (s, 2H, CH_2_), 6.81-6.84 (d, 2H, ArH, *J* = 6 Hz), 6.98-7.04 (t, 1H, ArH, *J* = 9 Hz), 7.19-7.27 (m, 1H, ArH), 7.31-7.34 (m, 1H, ArH), 7.40-7.43 (m, 1H, ArH), 7.83-7.86 (d, 2H, ArH, *J* = 9 Hz), 8.99 (s, 1H, =CH), 9.18 (s, 1H, OH), 9.19 (s, 1H, ArH); ^13^C NMR (DMSO-*d_6_*) δ (ppm) = 12.4 (CH_3_), 31.4 (3xCH_3_), 34.3 (C_t-butyl_), 45.8 (CH_2_), 46.9 (2xCH_2-piperazine_), 51.4 (2xCH_2-piperazine_), 56.0 (OCH_3_), 63.5 (CH_2_), 106.6, 113.5, 115.4, 125.5, 128.2, 128.6, 130.5, 131.1, 150.0, 150.1, 155.6 (15C_ar_), 157.6 (C_triazole_), 159.3, 159.8 (2C_ar_), 167.1 (C=O), 168.3 (C=S), 169.8 (C_ar_), 177.3 (C=O); Analysis for C_34_H_38_N_8_O_4_S (654.78) Calculated: C: 62.37%, H: 5.85%, N: 17.11%; Found: C: 62.44%, H: 5.79%, N: 17.06%.

***In vitro antimicrobial assays***

The examined compounds **3**-**50** were *in vitro* tested for antibacterial and antifungal activities with the use of the broth microdilution method according to European Committee on Antimicrobial Susceptibility Testing (EUCAST) [1] and Clinical and Laboratory Standards Institute guidelines [2] towards a panel of reference and clinical or saprophytic strains of microorganisms including Gram-positive bacteria (*Staphylococcus aureus* ATCC 25923, *Staphylococcus aureus* ATCC 43300, *Staphylococcus aureus* ATCC 6538, *Staphylococcus epidermidis* ATCC 12228, *Bacillus subtilis* ATCC 6633, *Bacillus cereus* ATCC 10876, *Micrococcus luteus* ATCC 10240), Gram-negative bacteria (*Bordetella bronchiseptica* ATCC 4617, *Escherichia coli* ATCC 25922, *Klebsiella pneumoniae* ATCC 13883, *Proteus mirabilis* ATCC 12453, *Salmonella typhimurium* ATCC 14028, *Pseudomonas aeruginosa* ATCC 9027) and fungi belonging to yeasts (*Candida albicans* ATCC 2091, *Candida albicans* ATCC 10231, *Candida parapsilosis* ATCC 22019, *Candida glabrata* ATCC 90030 and *Candida krusei* ATCC 14243). Antimicrobial activity assays were conducted according to the procedures described earlier by our group [3-5]. All the experiments were repeated three times (n = 3) and representative data is presented.

The statistical analysis of obtained results is presented in the Table 1S, 2S and 3S. Minimal Inhibitory Concentration (MIC) values in these tables are presented as Mode MIC - most frequent value in a data set of MIC values and mean MIC ± SE - standard error (SE) is the standard deviation of the sampling distribution of the mean of MIC values.

**References:**

1. European Committee for Antimicrobial Susceptibility Testing (EUCAST) (2003) determination of minimum inhibitory concentrations (MICs) of antibacterial agents by broth dilution. EUCAST discussion document E. Dis 5.1, Clin Microbiol Infect 9:1–7.

2. Clinical and Laboratory Standards Institute. Reference method for broth dilution antifungal susceptibility testing of yeasts. M27-S4. Clinical and Laboratory Standards Institute, Wayne, PA, USA, 2012.

3. Popiołek Ł, Biernasiuk A, Malm A (2015) Synthesis and antimicrobial activity of new 1,3-thiazolidin-4-one derivatives obtained from carboxylic acid hydrazides. Phosphorus Sulfur 190(2):251–260.

4. Popiołek Ł, Biernasiuk A (2016) Hydrazide-hydrazones of 3-methoxybenzoic acid and 4-tert-butylbenzoic acid with promising antibacterial activity against *Bacillus* spp. J Enzyme Inhib Med Chem 31(S1):62–69.

5. Popiołek Ł, Biernasiuk A (2016) Design, synthesis, and *in vitro* antimicrobial activity of hydrazide–hydrazones of 2-substituted acetic acid. Chem Biol Drug Des 88:873–883.

**Table 1S.** The activity data of compounds **3 - 18** expressed as mode MIC (mean MIC ± SE) [µg/ml] against the reference strains of bacteria and fungi.

‘-‘ – no activity; MIC – Minimal Inhibitory Concentration, n = 3 measurements; Mode MIC - most frequent value in a data set of MIC values; mean MIC ± SE - standard error (SE) is the standard deviation of the sampling distribution of the mean of MIC values. Compounds with bactericidal effect (MBC/MIC ≤ 4) are marked in bold.

| **Species** | | **Mode MIC (mean MIC ± SE) [µg/ml] of the tested compounds (n = 3)** | | | | |
| --- | --- | --- | --- | --- | --- | --- |
|  |  | **3** | **11** | **14** | **16** | **17** |
| **Gram-positive bacteria** | *Staphylococcus aureus*  ATCC 25923 | - | 1000  (1000.0 ± 0.0) | - | 1000  (1000.0 ± 0.0) | 250  (333.33 ± 144.34) |
|  | *Staphylococcus aureus*  ATCC 6538 | - | 1000  (1000.0 ± 0.0) | - | 1000  (1000.0 ± 0.0) | 125  (166.67 ± 72.17) |
|  | *Staphylococcus aureus*  ATCC 43300 | 1000  (1000.0 ± 0.0) | - | - | - | 1000  (1000.0 ± 0.0) |
|  | *Staphylococcus epidermidis*  ATCC 12228 | - | - | - | - | 250  (333.33 ± 144.34) |
|  | *Micrococcus luteus*  ATCC 10240 | - | 1000  (1000.0 ± 0.0) | - | 1000  (1000.0 ± 0.0) | 3.91  (5.21 ± 2.25) |
|  | *Bacillus subtilis*  ATCC 6633 | - | - | 1000  (1000.0 ± 0.0) | - | 31.25  (41.67 ± 18.04) |
|  | *Bacillus cereus*  ATCC 10876 | - | - | - | - | 7.81  (10.41 ± 4.51) |
| **Gram-negative bacteria** | *Bordetella bronchiseptica*  ATCC 4617 | 1000  (1000.0 ± 0.0) | 500  (666.67 ± 288.67) | - | - | 500  (666.67 ± 288.67) |
|  | *Klebsiella pneumoniae*  ATCC 13883 | - | - | - | - | 1000  (1000.0 ± 0.0) |
|  | *Proteus mirabilis*  ATCC 12453 | - | - | - | - | - |
|  | *Salmonella typhimurium*  ATCC 14028 | - | - | - | - | 1000  (1000.0 ± 0.0) |
|  | *Escherichia coli*  ATCC 25922 | - | - | - | - | 1000  (1000.0 ± 0.0) |
|  | *Pseudomonas aeruginosa*  ATCC 9027 | - | - | - | - | - |
| **Fungi** | *Candida albicans*  ATCC 2091 | **1000**  **(1000.0 ± 0.0)** | **250**  **(333.33 ± 144.34)** | - | - | 500  (666.67 ± 288.67) |
|  | *Candida albicans*  ATCC 10231 | **1000**  **(1000.0 ± 0.0)** | **250**  **(250.0 ± 0.0)** | - | - | 500  (500.0 ± 0.0) |
|  | *Candida parapsilosis*  ATCC 22019 | **1000**  **(1000.0 ± 0.0)** | **250**  **(333.33 ± 144.34)** | - | - | 250  (333.33 ± 144.34) |
|  | *Candida glabrata*  ATCC 90030 | 1000  (1000.0 ± 0.0) | **1000**  **(1000 ± 0.0)** | - | - | 1000  (1000.0 ± 0.0) |
|  | *Candida krusei*  ATCC 14243 | 1000  (1000.0 ± 0.0) | **250**  **(333.33 ± 144.34)** | - | - | 1000  (1000.0 ± 0.0) |

**Table 2S.** The activity data of compounds **19 - 34** expressed as mode MIC (mean MIC ± SE) [µg/ml] against the reference strains of bacteria and fungi.

‘-‘ – no activity; MIC – Minimal Inhibitory Concentration, n = 3 measurements; Mode MIC - most frequent value in a data set of MIC values; mean MIC ± SE - standard error (SE) is the standard deviation of the sampling distribution of the mean of MIC values. Compounds with bactericidal effect (MBC/MIC ≤ 4) are marked in bold.

| **Species** | | **Mode MIC (mean MIC ± SE) [µg/ml] of the tested compounds (n = 3)** | | | |
| --- | --- | --- | --- | --- | --- |
|  |  | **19** | **20** | **21** | **34** |
| **Gram-positive bacteria** | *Staphylococcus aureus*  ATCC 25923 | 1000  (1000.0 ± 0.0) | 1000  (1000.0 ± 0.0) | 500  (666.67 ± 288.67) | 1000  (1000.0 ± 0.0) |
|  | *Staphylococcus aureus*  ATCC 6538 | 1000  (1000.0 ± 0.0) | - | 500  (666.67 ± 288.67) | 500  (666.67 ± 288.67) |
|  | *Staphylococcus aureus*  ATCC 43300 | 1000  (1000.0 ± 0.0) | - | 1000  (1000.0 ± 0.0) | 1000  (1000.0 ± 0.0) |
|  | *Staphylococcus epidermidis*  ATCC 12228 | 1000  (1000.0 ± 0.0) | - | 1000  (1000.0 ± 0.0) | 1000  (1000.0 ± 0.0) |
|  | *Micrococcus luteus*  ATCC 10240 | 500  (666.67 ± 288.67) | 1000  (1000.0 ± 0.0) | 500  (500.0 ± 0.0) | 500  (666.67 ± 288.67) |
|  | *Bacillus subtilis*  ATCC 6633 | 500  (666.67 ± 288.67) | - | **500**  **(500.0 ± 0.0)** | 1000  (1000.0 ± 0.0) |
|  | *Bacillus cereus*  ATCC 10876 | 1000  (1000.0 ± 0.0) | 1000  (1000.0 ± 0.0) | 500  (666.67 ± 288.67) | 1000  (1000.0 ± 0.0) |
| **Gram-negative bacteria** | *Bordetella bronchiseptica*  ATCC 4617 | - | 1000  (1000.0 ± 0.0) | 1000  (1000.0 ± 0.0) | - |
|  | *Klebsiella pneumoniae*  ATCC 13883 | - | - | - | - |
|  | *Proteus mirabilis*  ATCC 12453 | - | - | - | - |
|  | *Salmonella typhimurium*  ATCC 14028 | - | - | - | - |
|  | *Escherichia coli*  ATCC 25922 | - | - | - | - |
|  | *Pseudomonas aeruginosa*  ATCC 9027 | - | - | - | - |
| **Fungi** | *Candida albicans*  ATCC 2091 | **250**  **(250.0 ± 0.0)** | **250**  **(333.33 ± 144.34)** | 500  (666.67 ± 288.67) | - |
|  | *Candida albicans*  ATCC 10231 | **250**  **(333.33 ± 144.34)** | **250**  **(333.33 ± 144.34)** | 500  (666.67 ± 288.67) | - |
|  | *Candida parapsilosis*  ATCC 22019 | **500**  **(500.0 ± 0.0)** | **500**  **(500.0 ± 0.0)** | 1000  (1000.0 ± 0.0) | - |
|  | *Candida glabrata*  ATCC 90030 | 500  (666.67 ± 288.67) | 1000  (1000.0 ± 0.0) | 1000  (1000.0 ± 0.0) | - |
|  | *Candida krusei*  ATCC 14243 | 1000  (1000.0 ± 0.0) | 1000  (1000.0 ± 0.0) | 1000  (1000.0 ± 0.0) | - |

**Table 3S.** The activity data of compounds **35 - 50** expressed as mode MIC (mean MIC ± SE) [µg/ml] against the reference strains of bacteria and fungi.

‘-‘ – no activity; MIC – Minimal Inhibitory Concentration, n = 3 measurements; Mode MIC - most frequent value in a data set of MIC values; mean MIC ± SE - standard error (SE) is the standard deviation of the sampling distribution of the mean of MIC values. Compounds with bactericidal effect (MBC/MIC ≤ 4) are marked in bold.

| **Species** | | **Mode MIC (mean MIC ± SE) [µg/ml] of the tested compounds (n = 3)** | | | | | | | | | | | | | | | |
| --- | --- | --- | --- | --- | --- | --- | --- | --- | --- | --- | --- | --- | --- | --- | --- | --- | --- |
|  |  | **35** | **36** | **37** | **38** | **39** | **40** | **41** | **42** | **43** | **44** | **45** | **46** | **47** | **48** | **49** | **50** |
| **Gram-positive bacteria** | *Staphylococcus*  *aureus*  ATCC 25923 | **31.25**  **(41.67 ± 18.04)** | **31.25**  **(31.25 ± 0.0)** | **62.5**  **(62.5 ± 0.0)** | **31.25**  **(41.67 ± 18.04)** | **62.5**  **(62.5 ± 0.0)** | 62.5  (83.3 ± 36.08) | **62.5**  **(83.3 ± 36.08)** | 125  (166.67 ± 72.17) | **125**  **(125.0 ± 0.0)** | **31.25**  **(41.67 ± 18.04)** | **31.25**  **(41.67 ± 18.04)** | **31.25**  **(31.25 ± 0.0)** | 125  (125.0 ± 0.0) | 62.5  (83.3 ± 36.08) | **125**  (166.67 ± 72.17) | **31.25**  **(41.67 ± 18.04)** |
|  | *Staphylococcus*  *aureus*  ATCC 6538 | **31.25**  **(31.25 ± 0.0)** | **15.62**  **(20.83 ± 9.02)** | 31.25  (31.25 ± 0.0) | 31.25  (31.25 ± 0.0) | **62.5**  **(62.5 ± 0.0)** | **62.5**  **(62.5 ± 0.0)** | **62.5**  **(62.5 ± 0.0** | **125**  **(166.67 ± 72.17)** | **125**  **(125.0 ± 0.0)** | **31.25**  **(41.67 ± 18.04)** | **31.25**  **(31.25 ± 0.0)** | **31.25**  **(31.25 ± 0.0)** | 62.5  (83.3 ± 36.08) | **62.5**  **(62.5 ± 0.0)** | **62.5**  **(83.3 ± 36.08)** | **15.62**  **(20.83 ± 9.02)** |
|  | *Staphylococcus*  *aureus*  ATCC 43300 | **500**  **(500.0 ± 0.0)** | **500**  **(500.0 ± 0.0)** | **500**  **(500.0 ± 0.0)** | **500**  **(500.0 ± 0.0)** | 125  (166.67 ± 72.17) | 500  (500.0 ± 0.0) | 500  (500.0 ± 0.0) | 250  (333.33 ± 144.34) | **500**  **(500.0 ± 0.0)** | **500**  **(500.0 ± 0.0)** | **500**  **(500.0 ± 0.0)** | 500  (500.0 ± 0.0) | 1000  (1000.0 ± 0.0) | 500  (500.0 ± 0.0) | 500  (500.0 ± 0.0) | 250  (333.33 ± 144.34) |
|  | *Staphylococcus*  *epidermidis*  ATCC 12228 | **31.25**  **(31.25 ± 0.0)** | **15.62**  **(15.62 ±**  **0.0)** | **31.25**  **(41.67 ± 18.04)** | **31.25**  **(41.67 ± 18.04)** | **31.25**  **(41.67 ± 18.04)** | **31.25**  **(41.67 ± 18.04)** | **31.25**  **(41.67 ± 18.04)** | **125**  **(125.0 ± 0.0)** | **62.5**  **(62.5 ± 0.0)** | **31.25**  **(41.67 ± 18.04)** | **31.25**  **(41.67 ± 18.04)** | **15.62**  **(41.67 ± 18.04)** | **62.5**  **(83.3 ± 36.08)** | **31.25**  **(41.67 ± 18.04)** | **62.5**  **(83.3 ± 36.08)** | **7.81**  (10.41 ± 4.51) |
|  | *Micrococcus*  *luteus*  ATCC 10240 | **250**  **(333.33 ± 144.34)** | **250**  **(333.33 ± 144.34)** | 250  (333.33 ± 144.34) | 250  (333.33 ± 144.34) | 250  (250.0 ± 0.0) | 500  (500.0 ± 0.0) | 250  (333.33 ± 144.34) | 250  (250.0 ± 0.0) | 250  (333.33 ± 144.34) | 250  (333.33 ± 144.34) | 250  (333.33 ± 144.34) | 250  (333.33 ± 144.34) | **500**  **(500.0 ± 0.0)** | 250  (333.33 ± 144.34) | 500  (500.0 ± 0.0) | 250  (250.0 ± 0.0) |
|  | *Bacillus*  *subtilis*  ATCC 6633 | **7.81**  **(7.81 ± 0.0)** | **3.91**  **(5.21 ± 2.25)** | **3.91**  **(5.21 ± 2.25)** | **7.81**  **(7.81 ± 0.0)** | **7.81**  **(7.81 ± 0.0)** | 7.81  (7.81 ± 0.0) | **7.81**  **(7.81 ± 0.0)** | 7.81  (10.41 ± 4.51) | **15.62**  **(15.62 ± 0.0)** | 7.81  (10.41 ± 4.51) | **3.91**  **(5.21 ± 2.25)** | **7.81**  **(7.81 ± 0.0)** | 7.81  (10.41 ± 4.51) | 7.81  **(7.81 ± 0.0)** | **31.25**  **(31.25 ± 0.0)** | **3.91**  **(5.21 ± 2.25)** |
|  | *Bacillus*  *cereus*  ATCC 10876 | 15.62  (15.62 ±  0.0) | 15.62  (15.62 ±  0.0) | 7.81  (10.41 ± 4.51) | 15.62  (20.83 ± 9.02) | 15.62  (20.83 ± 9.02) | 31.25  (31.25 ± 0.0) | 15.62  (20.83 ± 9.02) | 62.5  (62.5 ± 0.0) | 62.5  (62.5 ± 0.0) | 31.25  (31.25 ± 0.0) | 15.62  (20.83 ± 9.02) | 15.62  (15.62 ±  0.0) | 62.5  (62.5 ± 0.0) | 31.25  (41.67 ± 18.04) | 62.5  (83.3 ± 36.08) | 15.62  (15.62 ±  0.0) |
| **Gram-negative bacteria** | *Bordetella*  *bronchiseptica*  ATCC 4617 | **31.25**  **(41.67 ± 18.04)** | **31.25**  **(31.25 ± 0.0)** | **31.25**  **(31.25 ± 0.0)** | **31.25**  **(41.67 ± 18.04)** | **62.5**  **(62.5 ± 0.0)** | **62.5**  **(62.5 ± 0.0)** | **62.5**  **(62.5 ± 0.0)** | **125**  **(125.0 ± 0.0)** | **125**  **(125.0 ± 0.0)** | **31.25**  **(41.67 ± 18.04)** | **31.25**  **(41.67 ± 18.04)** | **31.25**  **(41.67 ± 18.04)** | **125**  **(125.0 ± 0.0)** | **62.5**  **(83.3 ± 36.08)** | **125**  **(166.67 ± 72.17)** | **31.25**  **(31.25 ± 0.0)** |
|  | *Klebsiella*  *pneumoniae*  ATCC 13883 | **15.62**  **(20.83 ± 9.02)** | **3.91**  **(5.21 ± 2.25)** | **7.81**  **(10.41 ± 4.51)** | **7.81**  **(10.41 ± 4.51)** | **15.62**  **(20.83 ± 9.02)** | **31.25**  **(41.67 ± 18.04)** | **31.25**  **(41.67 ± 18.04)** | **62.5**  **(62.5 ± 0.0)** | **62.5**  **(62.5 ± 0.0)** | **7.81**  **(10.41 ± 4.51)** | **15.62**  **(15.62 ± 0.0)** | **7.81**  **(10.41 ± 4.51)** | **62.5**  **(83.3 ± 36.08)** | **31.25**  **(41.67 ± 18.04)** | **62.5**  **(83.3 ± 36.08)** | **7.81**  **(10.41 ± 4.51)** |
|  | *Proteus*  *mirabilis*  ATCC 12453 | **1.95**  **(1.95 ± 0.0)** | **0.98**  **(1.30 ± 0.56)** | **3.91**  **(3.91 ± 0.0)** | **1.95**  **(2.60 ± 1.13)** | **1.95**  **(2.60 ± 1.13)** | **3.91**  **(5.21 ± 2.25)** | **3.91**  **(5.21 ± 2.25)** | **7.81**  **(10.41 ± 4.51)** | **7.81**  **(7.81 ± 0.0)** | **3.91**  **(5.21 ± 2.25)** | **3.91**  **(3.91 ± 0.0)** | **1.95**  **(2.60 ± 1.13)** | **7.81**  **(10.41 ± 4.51)** | **3.91**  **(5.21 ± 2.25)** | **7.81**  **(10.41 ± 4.51)** | **1.95**  **(2.60 ± 1.13)** |
|  | *Salmonella*  *typhimurium*  ATCC 14028 | **1.95**  **(1.95 ± 0.0)** | **1.95**  **(1.95 ± 0.0)** | **0.98**  **(0.98 ± 0.0)** | **0.98**  **(1.30 ± 0.56)** | **3.91**  **(3.91 ± 0.0)** | **3.91**  **(5.21 ± 2.25)** | **3.91**  **(5.21 ± 2.25)** | **7.81**  **(7.81 ± 0.0)** | **3.91**  **(5.21 ± 2.25)** | **3.91**  **(3.91 ± 0.0)** | **1.95**  **(1.95 ± 0.0)** | **1.95**  **(2.60 ± 1.13)** | **3.91**  **(5.21 ± 2.25)** | **3.91**  **(5.21 ± 2.25)** | **7.81**  **(7.81 ± 0.0)** | **1.95**  **(2.60 ± 1.13)** |
|  | *Escherichia*  *coli*  ATCC 25922 | **1.95**  **(1.95 ± 0.0)** | **0.98**  **(1.30 ± 0.56)** | **0.98**  **(1.30 ± 0.56)** | **0.98**  **(0.98 ± 0.0)** | **1.95**  **(2.60 ± 1.13)** | **3.91**  **(3.91 ± 0.0)** | **1.95**  **(2.60 ± 1.13)** | **3.91**  **(5.21 ± 2.25)** | **3.91**  **(5.21 ± 2.25)** | **1.95**  **(2.60 ± 1.13)** | **0.98**  **(1.30 ± 0.56)** | **1.95**  **(1.95 ± 0.0)** | **3.91**  **(5.21 ± 2.25)** | **1.95**  **(2.60 ± 1.13)** | **7.81**  **(10.41 ± 4.51)** | **0.98**  **(1.30 ± 0.56)** |
|  | *Pseudomonas*  *aeruginosa*  ATCC 9027 | **31.25**  **(41.67 ± 18.04)** | **31.25**  **(31.25 ± 0.0)** | **31.25**  **(41.67 ± 18.04)** | **31.25**  **(41.67 ± 18.04)** | **62.5**  **(62.5 ± 0.0)** | **125**  **(125.0 ± 0.0)** | **62.5**  **(62.5 ± 0.0)** | **125**  **(166.67 ± 72.17)** | **125**  **(125.0 ± 0.0)** | **31.25**  **(41.67 ± 18.04)** | **62.5**  **(62.5 ± 0.0)** | **31.25**  **(41.67 ± 18.04)** | **125**  **(125.0 ± 0.0)** | **62.5**  **(83.3 ± 36.08)** | **125**  **(166.67 ± 72.17)** | **31.25**  **(31.25 ± 0.0)** |
| **Fungi** | *Candida*  *albicans*  ATCC 2091 | - | - | - | 1000  (1000.0 ± 0.0) | - | - | - | 1000  (1000.0 ± 0.0) | 1000  (1000.0 ± 0.0) | - | - | - | - | - | - | - |
|  | *Candida*  *albicans*  ATCC 10231 | - | - | - | 1000  (1000.0 ± 0.0) | - | - | - | 1000  (1000.0 ± 0.0) | 1000  (1000.0 ± 0.0) | - | - | - | - | - | - | - |
|  | *Candida*  *parapsilosis*  ATCC 22019 | - | - | - | - | - | - | 1000  (1000.0 ± 0.0) | 500  (1000.0 ± 0.0) | - | - | - | - | - | - | 1000  (1000.0 ± 0.0) | - |
|  | *Candida*  *glabrata*  ATCC 90030 | - | - | - | - | - | - | 1000  (1000.0 ± 0.0) | 1000  (1000.0 ± 0.0) | - | - | - | - | - | - | - | - |
|  | *Candida*  *krusei*  ATCC 14243 | - | - | - | - | - | - | - | 1000  (1000.0 ± 0.0) | - | - | - | - | - | - | - | - |
